# Pro-Apoptotic Effects of Unsymmetrical Bisacridines in 3D Pancreatic Multicellular Tumor Spheroids

**DOI:** 10.3390/ijms26157557

**Published:** 2025-08-05

**Authors:** Agnieszka Kurdyn, Ewa Paluszkiewicz, Ewa Augustin

**Affiliations:** Department of Pharmaceutical Technology and Biochemistry, Faculty of Chemistry, Gdańsk University of Technology, Gabriela Narutowicza Str. 11/12, 80-233 Gdańsk, Poland; agnieszka.kurdyn@pg.edu.pl (A.K.); ewa.paluszkiewicz@pg.edu.pl (E.P.)

**Keywords:** unsymmetrical bisacridines, pancreatic cancer, multicellular tumor spheroids, apoptosis, anticancer activity

## Abstract

Pancreatic cancer (PC) is an aggressive malignancy with a poor prognosis, requiring innovative approaches to evaluate new therapies. Considering the high activity of unsymmetrical bisacridines (UAs) in PC monolayer cultures, we employed multicellular tumor spheroids (MCTS) to assess whether UAs retain pro-apoptotic activity under more physiologically relevant conditions. Ultra-low attachment plates were used to form spheroids from three PC cell lines (Panc-1, MIA PaCa-2, and AsPC-1) with different genotypes and phenotypes. The effects of UA derivatives (C-2028, C-2045, and C-2053) were evaluated using microscopy and flow cytometry (7-AAD for viability and annexin V-FITC/PI for membrane integrity). UAs altered the morphology of the spheroids and reduced their growth. Notably, Panc-1 spheroids exhibited compromised integrity. The increase in 7-AAD^+^ cells confirmed diminished cell viability, and annexin V-FITC assays showed apoptosis as the dominant death pathway. Interestingly, the exact derivative was most active against a given cell line regardless of culture conditions. These results confirm that UAs maintain anticancer activity in 3D cultures and induce apoptosis, with varying efficacy across different cell lines. This underscores the value of diverse cellular models in compound evaluation and supports UAs as promising candidates for pancreatic cancer therapy.

## 1. Introduction

Despite modern methods of cancer treatment and diagnosis, there is still a high demand for anticancer drugs with the highest possible efficacy and the lowest toxicity. Pancreatic cancer (PC) is notorious for its poor prognosis and high treatment resistance [1,2]. The overall five-year survival rate of less than 13% [3] reflects the aggressive nature of this cancer. Current treatment options, including surgery, chemotherapy, and radiation therapy, can extend survival time, but rarely provide a cure. The multidrug FOLFIRINOX (irinotecan, 5-fluorouracil, leucovorin, and oxaliplatin), which improves the median survival to 11.7 months, compared to 6.8 months with previous standard treatment with gemcitabine [4]. Recently, the FDA approved NALIRIFOX, which substitutes irinotecan with liposomal irinotecan, offering a new first-line therapy for metastatic PC [5]. However, pancreatic cancer exhibits pronounced resistance to standard therapies through several mechanisms. Its tumor microenvironment is marked by extensive desmoplastic stroma and poor vascularization, both of which hinder the delivery of drugs to cancer cells [6]. Additionally, pancreatic cancer cells often display intrinsic resistance via the overexpression of drug efflux pumps, such as multidrug resistance protein 1 (MDR1), also known as P-glycoprotein [7]. They also possess enhanced DNA damage repair mechanisms, altered apoptotic pathways, and activated pro-survival signaling pathways, including the PI3K/Akt and NF-κB pathways [8]. These factors collectively reduce the effectiveness of standard chemotherapy, contributing to rapid disease progression and recurrence. Consequently, overcoming chemoresistance remains a key goal in the development of new treatment strategies for pancreatic cancer.

**Figure 1:**
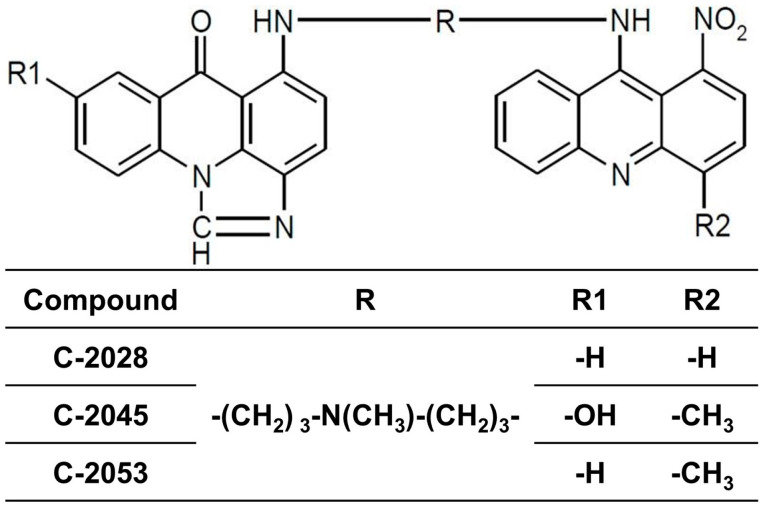
Chemical structures of tested unsymmetrical bisacridine derivatives: C-2028, C-2045, and C-2053.

Unsymmetrical bisacridine derivatives (UAs), as presented in Figure 1, are patented compounds with high anticancer properties that have been developed and widely studied by researchers from the Gdańsk University of Technology [9]. The chemical structure of UAs consists of an imidazoacridone and 1-nitroacridine moieties linked with an aminoalkyl chain (Figure 1). Extensive preliminary studies enabled the selection of three UA derivatives, C-2028, C-2045, and C-2053, which showed high activity against pancreatic cancer cells in particular [9]. Notably, UAs preferentially bind with G-quadruplex structures in oncogene promoters such as *KRAS* and *MYC* [9,10], rather than double-stranded DNA [11]. Modulation of c-Myc protein level by UAs resulted in apoptosis induction in colorectal (HCT116) and lung (H460) cancer cells [12]. Most importantly, this selective binding in pancreatic cancer monolayer (2D) cultures leads to the downregulation of the c-Myc protein level, inducing apoptosis, a process also facilitated by SMAD4 upregulation. In addition, UAs promote accelerated cellular senescence in MIA PaCa-2 cells by upregulating p21, with senescent cells eventually undergoing apoptosis following prolonged exposure [13]. Previous studies have also demonstrated the effectiveness of these compounds in three-dimensional (3D) culture models [14]. In colorectal (HCT116) and lung (A549) cancer cell-derived spheroids, UAs significantly inhibited spheroid growth and viability, confirming their ability to penetrate and act within the compact spheres. Further studies confirmed that UAs induce apoptosis in this culture model, consistent with their mechanism in monolayer cultures [14].

Considering the 2D culture results, this study aimed to test whether three UA derivatives—C-2028, C-2045, and C-2053—would exhibit similarly effective pro-apoptotic activity in PC cells cultured under 3D conditions. The application of multicellular tumor spheroids (MCTS) provides a robust and widely used platform for cancer research due to the diversity of methods used to generate them [15] and, most importantly, by more accurately representing the tumor microenvironment compared to traditional 2D cultures [16]. Spheroids consist of diverse zones defined by gradients of nutrients, oxygen, metabolites, waste, and pH, also present in poorly vascularized tumors [17]. MCTS are increasingly used for screening of new compounds, especially in PC cells that exhibit distinct morphological and functional characteristics in 3D cultures [18], which better reflect the complexity of this tumor biology [19].

The cell lines used in the current study, Panc-1, MIA PaCa-2, and AsPC-1, differ in their genetic profile [20] as well as the phenotype and expression of molecules responsible for cell–cell contacts [21]. Panc-1 and MIA PaCa-2 cells exhibit quasi-mesenchymal (epithelial and mesenchymal) features [22]. However, the Panc-1 cells express E-cadherin, reflecting a more epithelial-like phenotype, in contrast to MIA PaCa-2, which completely lacks this protein [23,24]. AsPC-1 cells are characterized by high levels of E-cadherin expression [23] and exhibit features of an intermediate phenotype between epithelial and mesenchymal states [25]. This morphological distinction within applied cell lines influences nutrient and oxygen gradients, affecting drug penetration and efficacy.

Since MCTS are more resistant to chemo- and radiotherapy compared to cells cultured in monolayers [26,27], this study examined whether UAs retain high anticancer activity against Panc-1, MIA PaCa-2, and AsPC-1 cells cultured under 3D conditions. To address this, we determined changes in the morphology, size, and viability of pancreatic cancer spheroids treated with UAs, as well as identified the main type of cellular response induced by these compounds.

## 2. Results

### 2.1. Optimization of 3D Culture Conditions

The first necessary step when starting research on a 3D cell culture model is to verify the ability of the tested cells to form multicellular tumor spheroids (MCTS), and if so, to determine the culture conditions. Generation of spheroids from cells at different densities using the ULA (ultra-low attachment) plates proved that the studied Panc-1, MIA PaCa-2, and AsPC-1 cells can form spheres (Figures S1–S3 in Supplementary Materials). Both Panc-1 and MIA PaCa-2 spheres exhibit similar morphometric features, such as a nearly spherical shape with jagged edges. In turn, AsPC-1 cell-derived spheroids were more condensed compared to the spheroids of the other tested lines, with an almost perfectly spherical shape and even edges. Moreover, the obtained spheroids exhibited a constant increase in size during incubation time and a high content of viable cells, which did not differ between spheroids, despite the varying numbers of cells from which they were generated. Cell viability on day 3 was assessed by flow cytometry using 7–AAD dye, and in Panc-1 cells, the content of 7–AAD^-^ (viable) cells ranged from 79.8% to 85.8%. In MIA PaCa-2 and AsPC-1 cell-derived spheroids, the percentage of viable cells was approximately 90%. Due to these observations, the spheroids of the studied cell lines are a promising culture model, and their steady growth and high viability of the cells that form MCTS allow further research on this model. Considering that the required spheroids diameter on day 0 (72 h after seeding) should be in the range of 300–500 µm [28], spheroids that were about 450 µm were used for further experiments, i.e., those generated from 1200 cells/well for Panc-1 cells, 500 cells/well for MIA PaCa-2 cells, and 4500 cells/well for AsPC-1 cells were used for further experiments.

### 2.2. Spheroid Size and Morphology Assessment After UAs Treatment

**Figure 2:**
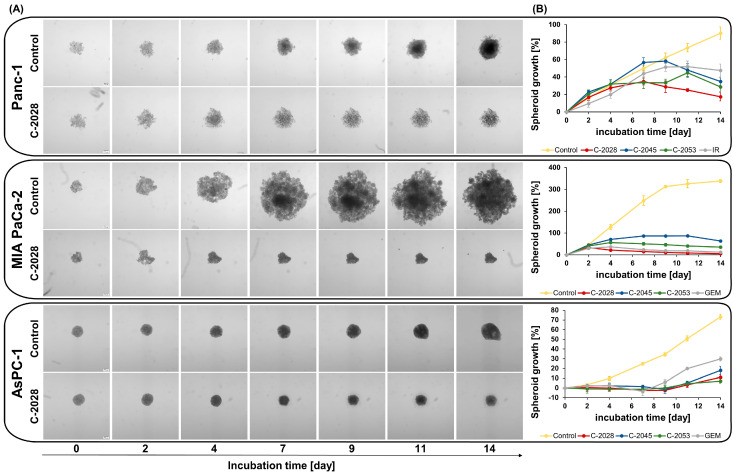
Morphology and kinetics of spheroids derived from pancreatic cancer cells after treatment with unsymmetrical bisacridine derivatives (UAs): C-2028, C-2045, and C-2053. Panc-1, MIA PaCa-2, and AsPC-1 spheroids were incubated with UAs at IC_80_ doses and positive control compounds (gemcitabine–GEM or irinotecan–IR) at IC_50_ doses for 14 days, and spheroid images were taken and their diameters were measured every 2–3 days. (**A**) Representative images of spheroids obtained from Panc-1, MIA PaCa-2, and AsPC-1 cells treated with C-2028 derivative. (**B**) Spheroid growth kinetics after exposure to UAs and positive control compounds are shown as spheroid growth compared to day 0 over time. Scale bar 200 μm. (*n* = 4).

The study of the effect that UAs induce in spheroids began with microscopic observation. Spheroids generated under the conditions determined in the previous paragraph, *Optimization of 3D culture conditions*, were exposed to UAs at a dose of IC_80_ and positive control compounds at a dose of IC_50_ (Table 1), and then subjected to 14 days of observation by taking photographs and diameter measurements every 2–3 days (Figure 2 and Figures S4–S6 in the Supplementary Materials). The selection of reference compounds and the doses of all compounds were based on previously conducted studies using pancreatic cancer cells cultured in 2D [13].

Control Panc-1 cell spheroids showed a steady increase in growth over time and became more compact and spherical. Treatment with the tested unsymmetrical bisacridines and irinotecan (IR) resulted in the morphology changes in the obtained spheroids (Figure 2 and Figure S4 in the Supplementary Materials). After only 4 days of incubation, compound-treated spheroids became loose with irregular edges, and the cells forming the spheroids shrank. Spheroids treated with the C-2028 derivative showed the strongest growth inhibition, only a 17% increase, while C-2045 and irinotecan led to more diffuse spheroids with 35% and 47% increases, respectively, after 14 days of incubation.

Control spheroids generated from MIA PaCa-2 cells (Figure 2) exhibited rapid growth, with a growth rate of 338% after 14 days (Figure S5 in the Supplementary Materials). Their structure was heterogeneous, characterized by areas of higher cell density and jagged, uneven edges. Treatment of the spheroids with the compounds significantly reduced their proliferative capacity, resulting in them being much smaller than the control spheroids. The spheroids exposed to the C-2028 derivative were the most compact. They were characterized by the smallest diameter already on the fourth day of incubation—a 21% increase in size compared to day 0 (Figure S5 in the Supplementary Materials). Treatment with C-2045 and C-2053 resulted in reduced proliferation and the formation of loose, irregular spheroids. After 14 days, their diameters increased by 63% and 35%, respectively, whereas C-2028 showed the most effective growth inhibition, with only a 5% increase in diameter. Treatment with gemcitabine (GEM), as with the C-2028 derivative, caused shrinkage of the spheroids, except that this occurred after the seventh day of incubation, and their structure was less compact.

The morphology of the spheroids generated from AsPC-1 cells (Figure 2 and Figure S6 in the Supplementary Materials), not treated with the compounds, was homogeneous, and they maintained their spherical shape even up to the 11th day of incubation. Compared to MIA PaCa-2 spheroids, those obtained from AsPC-1 cells showed slower and more restricted growth. Treatment of AsPC-1 spheroids with UAs and GEM resulted in morphology changes after the seventh day of exposure (Figure S6 in the Supplementary Materials). The spheroids had a smooth periphery up to day 4 of incubation, while a diffuse outer layer of cells was observed from day 7 onward. All UAs similarly reduced the spheroid growth after 14 days (7–18%) (Figure S6 in the Supplementary Materials). In contrast, gemcitabine caused loss of spheroid integrity with the largest diameter increase among the tested compounds (30%) (Figure S6 in the Supplementary Materials). In general, C-2028 showed the most substantial growth inhibitory effect in all cell lines.

### 2.3. The Impact of UAs on Cell Viability in 2D and 3D

**Figure 3:**
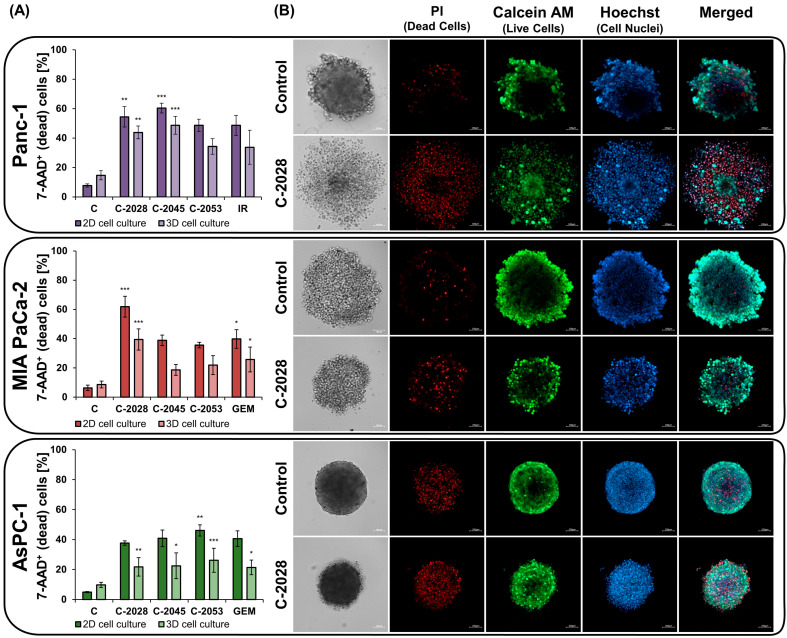
Viability of pancreatic cancer cells cultured in 2D and 3D conditions after treatment with unsymmetrical bisacridine derivatives (UAs): C-2028, C-2045, and C-2053. Panc-1, MIA PaCa-2, and AsPC-1 cells were incubated with UAs at IC_80_ doses and positive control compounds (gemcitabine–GEM or irinotecan–IR) at IC_50_ doses for 72 h, followed by viability analysis using flow cytometry (2D and 3D) and imaging using confocal microscopy (3D). (**A**) Bar graphs show the flow cytometry-quantified percentage of dead (7-AAD^+^) cells in 2D and 3D cultures after exposure to the tested compounds. (**B**) Representative images of spheroids treated with C-2028 derivative and stained with propidium iodide (PI), calcein AM, and Hoechst 33342. Scale bar 100 μm. Statistical analysis was performed using the Kruskal–Wallis test followed by Dunn’s post hoc test. Differences versus control: * *p*  <  0.05, ** *p*  <  0.01, *** *p*  <  0.001. (*n* ≥ 2).

Exposure to UAs altered MCTS morphology, indicating reduced cell proliferation and induction of cell death. To determine viability, cells were cultured under 2D and 3D conditions and then treated for 72 h with UAs at IC_80_ and positive control compounds at IC_50_. The cells were then stained with 7–AAD, which binds to the DNA of damaged cells, and subjected to flow cytometry analysis (Figure 3A and Tables S1 and S2 in Supplementary Materials).

In monolayer culture, Panc-1 cells treated with UAs for 3 days exhibited a higher percentage of dead cells compared to the untreated control (Figure 3A and Table S1 in the Supplementary Materials). Among the compounds tested, C-2045 induced cell death to the greatest extent (60.4%, *p* < 0.001, Figure 3A), while C-2028 and C-2053 caused a moderate effect (48.6–54.5% of 7-AAD^+^ cells, Table S1 in the Supplementary Materials). In contrast, the spherical culture initially had a lower content of viable cells compared to the 2D culture, and the viable cell content in the control spheroids reached 85.3% after 72 h of incubation (Figure 3A and Table S2 in the Supplementary Materials). The Panc-1 cells cultured in 3D conditions were also the most sensitive to the C-2045 derivative, inducing death in 48.7% (*p* < 0.001, Figure 3A) of the cells. The percentage of 7-AAD^+^ cells after exposure to the C-2028 derivative was 43.8% (*p* < 0.01, Figure 3A), while C-2053 and IR had a very similar effect of 34.3–33.7% non-viable cells (Table S2 in the Supplementary Materials).

MIA PaCa-2 cells cultured in 2D conditions were characterized by a high content of alive cells—93.6% (Figure 3A and Table S1 in the Supplementary Materials). Exposure to UAs significantly increased the 7-AAD^+^ dead cell population to the greatest extent for the C-2028 derivative—61.8% (*p* < 0.001, Figure 3A). The percentage of dead cells after treatment with C-2045, C-2053, and GEM was similar, reaching 38.8%, 35.6%, and 39.8%, respectively (*p* < 0.05, Figure 3A). Untreated cells cultured in both 3D and 2D conditions also showed a high content of viable cells—90.4% (Figure 3A and Table S2 in the Supplementary Materials). Similarly to the 2D culture, C-2028 was the most potent in spheroid culture, inducing cell death in 39.4% of cells (*p* < 0.001, Figure 3A), followed by C-2045, C-2053, and GEM with a non-viable cell population in the range of 18.7–25.8% (Table S2 in the Supplementary Materials).

AsPC-1 cells cultured as monolayers also demonstrated high viability, reaching 95% of the cell population (Figure 3A and Table S1 in the Supplementary Materials). Exposure of the cells to UAs and GEM increased the dead cell population to a range of 37.7–46.1% (Table S1 in the Supplementary Materials), and the best effect was produced by the C-2053 derivative—46.1% (*p* < 0.01, Figure 3A). Control spheroids were characterized by a high level of viable cells, 90.3%, and exposure to the test compounds, as in 2D cultures, caused an effect at a similar level (Figure 3A and Table S2 in the Supplementary Materials). The C-2028 derivative induced death in 21.7% (*p* < 0.01, Figure 3A) of cells, C-2045—22.5% (*p* < 0.05, Figure 3A), and GEM—21.4% (*p* < 0.05, Figure 3A). As in 2D culture, C-2053 proved to be the most active against AsPC-1 spheroids, resulting in the death of 26.1% of the cell population (*p* < 0.001, Figure 3A).

To visualize and confirm the results of cytometric analysis for 3D cultures, spheroid imaging was performed after 72 h of compound treatment (Figure 3B and Figures S7–S9 in the Supplementary Materials). Hoechst 33342 was used to determine the total cell count, while calcein AM marked only metabolically active cells. Propidium iodide (PI) was used to distinguish between dead cells with compromised membranes and live cells.

As shown in Figure 3B, control spheroids Panc-1, MIA PaCa-2, and AsPC-1 showed mainly calcein AM-positive cells. Green staining was predominantly localized in the outer layers of the 3D structure, where cells have easier access to nutrients and oxygen. Weak PI positivity was observed, indicating that only a small percentage of cancer cells forming spheroids underwent cell death. In contrast, spheroids treated with UAs and positive control compounds (Figure 3B and Figures S7–S9 in the Supplementary Materials) exhibited an increase in PI fluorescence accompanied by a decrease in the number of calcein-positive cells. As in the control, in compound-treated spheroids, calcein AM-positive cells were present mainly at the periphery of their structure. In contrast, cells labeled with propidium iodide were observed throughout the spheroid structure. These observations demonstrate the induction of cell death in the spheroids exposed to UAs and confirm the results of cytometric analysis.

### 2.4. AnnexinV–FITC/PI Double Staining

**Figure 4:**
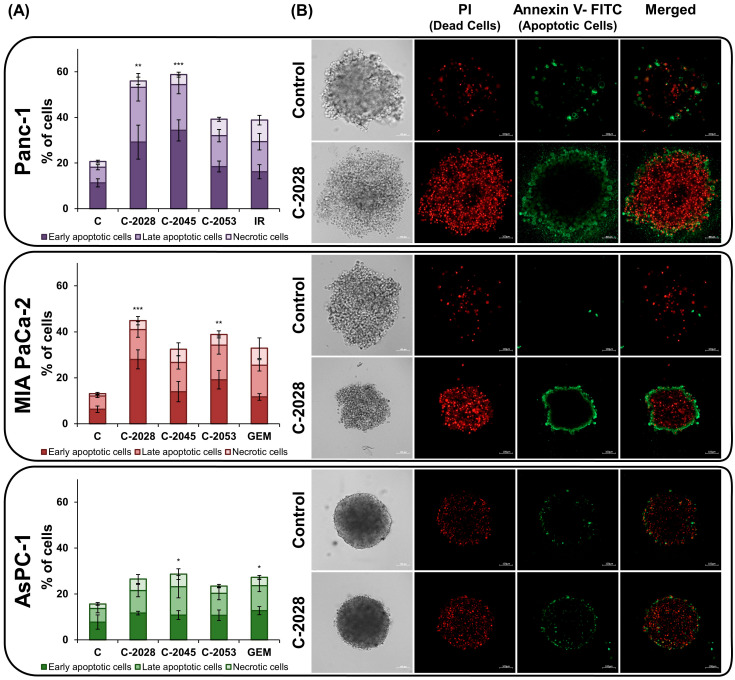
Phosphatidylserine externalization and membrane disruption of pancreatic cancer cells cultured in 3D conditions after treatment with unsymmetrical bisacridine derivatives (UAs): C-2028, C-2045, and C-2053. Panc-1, MIA PaCa-2, and AsPC-1 spheroids were incubated with UAs at IC_80_ doses, and positive control compounds (gemcitabine–GEM or irinotecan–IR) at IC_50_ doses for 72 h, followed by propidium iodide (PI)/annexin V–fluorescein isothiocyanate (FITC) dual staining. Early apoptotic cells—annexin V–FITC positive, PI negative; late apoptotic cells—annexin V–FITC positive, PI positive; necrotic cells—annexin V–FITC negative, PI positive. (**A**) Bar graphs show the percentage of early apoptotic, late apoptotic, and necrotic cells in 3D culture after exposure to the tested compounds. (**B**) Representative images of spheroids treated with C-2028 derivative and stained with PI and annexin V–FITC. Scale bar 100 μm. Statistical analysis was performed using the Kruskal–Wallis test followed by Dunn’s post hoc test. Differences versus control: * *p*  <  0.05, ** *p*  <  0.01, *** *p*  <  0.001. (*n* ≥ 2).

Considering that UAs cause cell death in spheroids and that the main type of death induced under 2D conditions was apoptosis [13], the next step was to test whether the same kind of cell death would occur in spheroids. Accordingly, spheroids were incubated for 72 h with UAs at IC_80_ doses and reference compounds at IC_50_ doses. Then, the cells were stained with annexin V–FITC/PI and subjected to flow cytometry analysis (Figure 4A and Table S3 in the Supplementary Materials).

Cytometric analysis showed an increase in the population of dead cells, mainly apoptotic cells, in UA-treated spheroids compared to controls (Figure 4A and Table S3 in the Supplementary Materials). A slight predominance of early-apoptotic cells over late-apoptotic cells was observed for most compounds studied; however, this difference reached statistical significance only in Panc-1 spheroids treated with C-2045 (*p* < 0.05). Necrosis was induced in a small percentage of all cells tested in 3D culture. For UAs, the population of PI-positive cells did not exceed 7.2%, whereas for reference compounds, it reached a maximum of 9.5%.

Spheroids generated from Panc-1 and MIA PaCa-2 cells, which were characterized by a less compact structure, appeared to be more sensitive to UA derivatives compared to AsPC-1 spheroids. Panc-1 spheroids were similarly susceptible to C-2028 and C-2045; apoptosis was induced in 53.2 and 54.0% of the cell population, respectively (Table S3 in the Supplementary Materials). Total number of dead cells, including necrosis, was 56.0% (*p* < 0.01, Figure 4A) for C-2028 and 58.4% (*p* < 0.001, Figure 4A) for C-2045. The C-2045 induced the greatest disproportion between early (34.6%) and late (20.1%) apoptosis. C-2053 was less effective (32.0% apoptotic cells), while irinotecan induced apoptosis in only 29.4% of cells (Table S3 in the Supplementary Materials). In MIA PaCa-2 cells, exposure to the C-2028 derivative resulted in phosphatidylserine translocation in the highest percentage of cells—41.0%, of which 28.0% were early apoptotic cells, and the total number of dead cells was 44.8% (*p* < 0.001, Figure 4A). C-2053 caused a weaker effect, resulting in 34.2% apoptotic cells, and when including necrotic cells, a total of 38.9% (*p* < 0.01, Figure 4A), whereas C-2045 and GEM induced cell death by apoptosis at similar levels, 26.6 and 25.4% (Table S3 in the Supplementary Materials). AsPC-1 spheroids were more resistant, with a comparable degree of apoptosis induction across all compounds, ranging from 20.3% to 23.7% (Table S3 in the Supplementary Materials). C-2045 led to 23.2% of apoptotic cells, and including necrosis, a total of 28.7% (*p* < 0.05, Figure 4A) dead cells. In comparison, GEM had an equivalent effect, inducing 23.7% (Table S3 in the Supplementary Materials) apoptotic cells and a total of 27.3% (*p* < 0.05, Figure 4A) dead cells. In turn, C-2028 and C-2053 triggered early and late apoptosis in 21.5% and 20.3% of cells, respectively (Table S3 in the Supplementary Materials).

Spheroid imaging was used to detect early- and late-apoptotic cells, as well as necrotic cells, after 72 h of compound treatment. As in the cytometric analysis, annexin V–FITC stained apoptotic cells, while PI stained necrotic and late apoptotic cells (Figure 4B).

As shown in Figure 4B and Figures S10–S12 in the Supplementary Materials, the fraction of apoptotic and necrotic cells was significantly lower in untreated spheroids of all tested cell lines, compared to spheroids exposed to UA compounds. In the merged images, especially of compound-treated spheroids, it is possible to distinguish between early-apoptotic cells, whose cell membranes were stained green with annexin V–FITC, and late-apoptotic cells, which were stained with both annexin V–FITC and propidium iodide. Accordingly, late-apoptotic cells are observed as red-stained interiors where PI has bound to DNA, surrounded by a green envelope, containing red-stained DNA inside. These observations qualitatively confirm the results of the cytometric analysis—the induction of apoptosis in spheroids formed from pancreatic cancer cells.

## 3. Discussion

The results presented in the current study on multicellular tumor spheroids derived from Panc-1, MIA PaCa-2, and AsPC-1 cells provide critical insights into the efficacy of three highly active unsymmetrical bisacridine derivatives. UAs significantly altered the morphology of treated spheroids and reduced their growth. These changes were caused by the induction of cell death, particularly apoptosis, demonstrating the anticancer nature of the tested compounds against pancreatic cancer cells cultured under 3D conditions.

Our recently published work proved that UAs exhibit high anticancer potential against pancreatic cancer cells cultured as monolayers [13]. Given the increasing popularity of the three-dimensional culture model, studies were conducted to evaluate the effect of UAs on cells with different phenotypes, specifically Panc-1, MIA PaCa-2, and AsPC-1, cultured under 3D conditions. The use of a 3D culture model has many advantages, including a closer representation of in vivo conditions; however, anticancer compounds may appear effective in 2D but lose potency in a more biologically relevant 3D environment [29]. Therefore, the present study aimed to determine whether UA derivatives retain potent pro-apoptotic efficacy under more physiologically relevant conditions.

Morphological distinction within applied cell lines can affect drug penetration and efficacy. As a result, loose spheroids may allow for better diffusion of therapeutic agents compared to compact spheroids, potentially leading to varied responses to treatment across different cell lines [16]. Such phenomena were observed in our study. Tested UAs inhibited spheroid growth to the greatest extent in highly proliferative MIA PaCa-2 cells, with C-2028 being the most active derivative. The less dense structure of these spheres with jagged edges allowed for reaching the interior of the spheroid, thus facilitating the compound action. In most dense AsPC-1 cell-derived spheroids, all UAs inhibited growth more than GEM, with C-2053 showing the most substantial effect. A different impact from that observed in MIA PaCa-2 and AsPC-1 occurred in Panc-1 spheres treated with the compounds. In addition to a reduction in the size of spheroids exposed to compounds, a significant dispersion of their structure was observed, mainly after C-2028 treatment. Inhibition of the growth of the spheroid as well as loss of its integrity under the influence of the anticancer compound are the most frequently observed changes, which strictly depend on the mechanism of action of the compounds [30], but also on the cell lines applied. In our previous studies, after using the same UA derivatives, a reduction in the size of A549 spheroids, along with an incoherent layer of peripheral cells, was observed in HCT116 spheroids [14]. Notably, the spheroids of lung and colon cancers had a distinct morphology from the spheroids presented in this study, most closely resembling those observed for AsPC-1 cells.

Alternations of spheroids’ morphology after treatment with UAs indicated a reduction in cell viability and proliferation; therefore, cytometric 7–AAD viability analysis was conducted. Since cells in 3D cultures can exhibit drug resistance and different survival characteristics, making them less susceptible to cytotoxic compounds compared to those grown in 2D [31,32], here we assessed the distribution of pancreatic cancer cell viability cultured under 2D and 3D conditions. Firstly, untreated cells cultured as a monolayer exhibited a lower population of dead cells compared to 3D conditions. This distinction can be explained by the gradients of oxygen and nutrients that can lead to a necrotic core in the three-dimensional structure of spheroids. At the same time, monolayer cultures do not experience such gradients [33]. Most importantly, the results proved that UAs reduced the population of viable cells in 3D compared to the control. In addition, cells cultured under both conditions and exposed to UA were most sensitive to the exact derivative: C-2045 for Panc-1, C-2028 for MIA PaCa-2, and C-2053 for AsPC-1. Positive control drugs caused less cell death than at least one UA derivative in all cells. Moreover, Panc-1 cell-derived spheroids were the most sensitive to UAs, likely due to the UA-induced loss of spheroid integrity, which facilitates the diffusion of drugs. Fluorescence staining confirmed decreased viability and membrane integrity after UA exposure, as evidenced by the increase in PI-positive cells along with the decrease in calcein AM-positive cells [34,35]. Calcein also indicated uptake, efflux, and inward diffusion within spheroids, revealing the dynamics of the drug in a 3D environment [36]. The peripheral fluorescence suggested active efflux transporter activity, such as MDR1 [37,38]. Although UAs are MDR1 substrates, they do not affect the expression of ABCB1, ABCC1, and ABCC2 genes in certain cancer cells, suggesting a lower risk of developing resistance and highlighting their strong anticancer potential [39].

Identification of the type of cell death induced by UAs using annexin V-FITC and propidium iodide staining indicated apoptosis, with a low level of necrosis observed. Moreover, the early-apoptotic cell fraction was slightly higher in spheroids exposed to compounds, although statistical significance was achieved only in Panc-1 spheroids treated with C-20245. In contrast, as reported in our previous work [13], after 72 h of compound treatment, the late-apoptotic cell fraction prevailed in 2D. This shift suggests delayed compound action by limited penetration through the spheroid structure [40]. Nevertheless, all the UA derivatives studied had high activity in 3D, sometimes slightly better than in 2D [13]. For instance, C-2028 caused more cell death in Panc-1 spheroids (56%) than in 2D (42.5%). Similarly, C-2045 was about 5% more active in 3D across all cell lines, and C-2028 caused nearly 14% more death in MIA PaCa-2 spheroids than in 2D [13]. However, it is worth noting that baseline cell death was higher in untreated spheroids than in monolayers.

The high potential of UAs in pancreatic MCTS may be related to the acidic spheroid microenvironment [41], which enhances UAs’ solubility, uptake, and stability. Our previous studies indicate that UAs exhibit enhanced anticancer activity under acidic conditions due to the protonation of functional groups at lower pH, which increases their solubility and promotes interactions with cancer cell membranes, thereby improving cellular uptake [42]. Conversely, positive controls (IR or GEM) performed worse in 3D, aligning with other studies reporting diminished GEM efficacy in MIA PaCa-2 spheroids [27], whereas a common approach in the literature to overcome the resistance of MIA PaCa-2 and AsPC-1 spheroids to GEM is its administration in combination with other anticancer compounds [40,43]. Although the search for new combination therapies with gemcitabine is still ongoing to potentially increase efficacy against pancreatic cancer, it is essential to note that its use may carry risks related to toxicity and the development of resistance, leading to only marginal survival benefits [44].

Summing up, this study highlights the pro-apoptotic potential of UAs against PC cells, a notoriously drug-resistant malignancy. The morphological differences among spheroids of Panc-1, MIA PaCa-2, and AsPC-1 cells, along with their varied responses to UA treatment, underscore the importance of three-dimensional models in evaluating anticancer compounds. Based on our previous studies using a 2D pancreatic cancer culture model, UAs were found to induce apoptosis by downregulating the c-Myc protein, the primary molecular target of these compounds [13]. Although the molecular profile in 3D cultures may differ from that observed in 2D, it is promising that UAs triggered a similar final cellular response while sustaining robust anticancer activity, even in the more challenging 3D spheroids. These findings lay the groundwork for future research on unsymmetrical bisacridines in more advanced 3D models and, subsequently, in animal models, supporting their continued evaluation as candidates for pancreatic cancer therapy.

## 4. Materials and Methods

### 4.1. Tested Compounds

Three unsymmetrical bisacridine derivatives (UAs), C-2028, C-2045, and C-2053, were synthesized as hydrochlorides at the Department of Pharmaceutical Technology and Biochemistry, Gdańsk University of Technology, according to the procedure previously published [9]. Stock solutions of the UAs were prepared in sterile, deionized Milli-Q water (Merck/Millipore, Burlington, MA, USA). The positive control drugs, gemcitabine and irinotecan (Merck/Sigma-Aldrich, St. Louis, MO, USA), were prepared as stock solutions in sterile, deionized Milli-Q water and dimethyl sulfoxide (DMSO; POCH S.A., Gliwice, Poland), respectively. Working solutions of all compounds were prepared in sterile, deionized Mili-Q water. The selection of reference compounds and the doses of all compounds were based on previously conducted studies using pancreatic cancer cells cultured in 2D [13].

### 4.2. Cell Lines and Culture Conditions

Three human pancreatic cancer cell lines, Panc-1 (CRL-1469), MIA PaCa-2 (CRL-1420), and AsPC-1 (CRL-1682), were purchased from the American Type Culture Collection (ATCC, Manassas, VA, USA). Panc-1 and MIA PaCa-2 cells were maintained in high-glucose Dulbecco’s modified Eagle’s medium (DMEM HG, Merck/Sigma-Aldrich, St. Louis, MO, USA). In contrast, AsPC-1 cells were maintained in RPMI 1640 medium (Merck/Sigma-Aldrich, St. Louis, MO, USA). Both media were supplemented with heat-inactivated 10% fetal bovine serum (FBS), 100 μg/mL streptomycin, and 100 units/mL penicillin. Cells were maintained in a Falcon 75 cm^2^ rectangular, canted-neck cell culture flask with a vented cap (Corning Incorporated, Kennebunk, ME, USA) and incubated in a humidified atmosphere containing 5% CO_2_ at 37 °C. All experiments were performed on cells in a logarithmic growth phase.

### 4.3. Generation of Multicellular Tumor Spheroids

Multicellular tumor spheroids (MCTS) were generated using Corning^®^ Costar^®^ Ultra-Low Attachment (ULA) 96-well round-bottom plates (Corning Incorporated, Kennebunk, ME, USA). A cell density gradient was used to determine the number of cells used to generate spheroids, selecting those that formed spheroids with a diameter of about 450 μm on day 3 after seeding. During optimization, the following spheroid generation conditions were selected: Panc-1-1200 (cells/well), MIA PaCa-2-500 (cells/well), and AsPC-1-4500 (cells/well). Briefly, to generate MCTS from monolayer cultures, cells were trypsinized, counted, and centrifuged to remove trypsin. Then, cells were resuspended in fresh medium at a density suitable for spheroid formation. Next, 200 µL/well of cell suspension was added to each well of a ULA plate, which was centrifuged for 15 min at 1200 rpm (A-4-62 rotor) at room temperature to initiate aggregation. Plates were incubated at 37 °C in a humidified atmosphere with 5% CO_2_ for 3 days. On day 3 (designated as day 0), 100 µL of the medium was replaced with fresh medium. Spheroids were imaged using a ×4 objective on an Olympus IX83 inverted microscope (Olympus, Tokyo, Japan) with an XC50 camera (Olympus, Tokyo, Japan) and CellSens Dimension software version 1.18 (Olympus, Tokyo, Japan).

### 4.4. Spheroid Size and Morphology Assessment

The pancreatic cancer MCTS were generated as described in the previous paragraph *Generation of Multicellular Tumor Spheroids* and photographed at day 0; next, 100 µL of culture medium was replaced with a fresh medium in the control spheroids or a fresh medium with IC_80_ concentrations of UAs and IC_50_ concentrations of positive control in the treated spheroids. Images of at least 8 spheroids grown under given conditions were taken every 2–3 days (up to 14 days). Along with the photos taken, the diameters of the spheroids were measured using the cellSens Dimension software version 1.18. The kinetics of spheroid size change were expressed as the ratio of the mean spheroid diameter value on the day of measurement (*d_x_*) minus the mean spheroid diameter value on day 0 (*d_0_*) to the *d_0_* value, according to the formula below. Results were obtained from four independent experiments (*n* = 4).

$$pheroid\;growth\;\left[\%\right]=\frac{d_{x}-d_{0}}{d_{0}}\cdot 100\%$$

### 4.5. Cell Death Assay

The cell death assay was performed using 7-aminoactinomycin D (7–AAD) dye (Thermo Scientific, Waltham, MA, USA). Briefly, pancreatic cancer cells cultured as monolayers were seeded at a density of 1.5 × 10^6^ for Panc-1 and 1 × 10^6^ for MIA PaCa-2 and AsPC-1 per Falcon 100 mm cell culture dish (Corning Incorporated, Kennebunk, ME, USA). Cells were then treated with IC_80_ doses of UAs and IC_50_ doses of positive controls for 72 h. Following exposure to the test compounds, 0.5 × 10^6^ cells were collected from the plates, centrifuged at 1000 rpm (A–4–62 rotor) for 5 min at RT, and washed twice with cold PBS. Next, cells were resuspended in 150 µL of PBS and stained with 7–AAD dye (1 µg/mL) for 15 min in the dark at RT. In turn, the pancreatic cancer MCTS were generated as described in the paragraph *Generation of Multicellular Tumor Spheroids*, and on day 0, 100 µL of culture medium was replaced with a fresh medium in the control spheroids or a fresh medium with IC_80_ concentrations of UAs and IC_50_ concentrations of positive control in the treated spheroids. After compound exposure, the spheroids (16–48 spheroids, dependent on cell line) were collected and trypsinized to obtain a single-cell suspension. Next, a fresh medium was added to neutralize the trypsin, and cells were centrifuged, washed twice with PBS, resuspended in 150 µL of PBS, and stained with 1 µg/mL 7–AAD for 15 min in the dark at RT. After staining, samples were analyzed using a FACS AccuriTM C6 (BD, San Jose, CA, USA) flow cytometer. At least 10,000 cells were collected, and the results were analyzed using BD AccuriTM C6 Software Version 1.0.264.21. Results were obtained from four independent experiments (*n* = 4).

### 4.6. Annexin V/PI Dual Staining

Apoptosis detection was performed by analyzing changes in the cytoplasmic membrane using flow cytometry with the FITC Annexin V Apoptosis Detection Kit (BD Biosciences, San Jose, CA, USA) according to the manufacturer’s instructions. Briefly, spherical cultures of Panc-1, MIA PaCa-1, and AsPC-1 cells were exposed to UAs and a positive control for 72 h and harvested as described in the previous paragraph *Cell Death Assay*. Cell pellets were resuspended in 50 µL of a binding buffer containing Annexin V–FITC and PI and incubated for 15 min in the dark at RT. Next, the samples were diluted with 180 µL of binding buffer and analyzed using an FACS Accuri C6 flow cytometer (BD, San Jose, CA, USA). At least 10,000 cells were collected, and the results were analyzed using BD AccuriTM C6 Software Version 1.0.264.21. Results were obtained from four independent experiments (*n* = 4).

### 4.7. Spheroid Staining

Panc-1, MIA PaCa-2, and AsPC-1 spheroids were exposed for 72 h to UAs and positive controls as described in the paragraph *Cell Death Assay*. Following incubation, spheroids were stained using either (1) Hoechst 33342, calcein AM, and propidium iodide (PI), or (2) Annexin V-FITC and PI dye mixtures. Calcein AM (Invitrogen, Thermo Scientific, Waltham, MA, USA) and Hoechst 33342 (Sigma-Aldrich, St. Louis, MO, USA) were obtained in lyophilized form and reconstituted according to the manufacturers’ instructions to yield 1 mg/mL stock solutions in DMSO and ultrapure water, respectively. For each spheroid suspended in 200 µL of culture medium, 0.3 µL of calcein AM and 0.5 µL of Hoechst 33342 stock solution were added, resulting in final concentrations of 1.5 µg/mL and 2.5 µg/mL, respectively. PI and Annexin V-FITC (BD Biosciences, San Jose, CA, USA) were purchased as ready-to-use liquid formulations. For each well, 0.8 µL of PI and 0.8 µL of Annexin V-FITC were added. Dye mixtures were prepared immediately before use in bulk for the total number of samples. Then, 1.6 μL of the mixed dye solution was added to each well containing a spheroid, and the samples were incubated for 2–3 h at 37 °C in a humidified atmosphere with 5% CO_2_ before imaging. To avoid spheroid disintegration, staining was performed in complete culture medium without washing steps. Image sections were acquired at 15 µm intervals using an LSM 800 inverted laser scanning confocal microscope (Carl Zeiss, Jena, Germany), equipped with an Airyscan detector for high-resolution confocal scanning, and a ×10 objective (Carl Zeiss, Jena, Germany). Images were processed with ZEN 2.6 software (Carl Zeiss, Jena, Germany). Results were obtained from two independent experiments (*n* = 2).

### 4.8. Statistical Analysis

The results are presented as the means ± SDs from at least three independent experiments. The normality of data was assessed using the D’Agostino–Pearson test. Statistical analysis was performed using the Kruskal–Wallis one-way analysis of variance for non-parametric data and Dunn’s multiple comparison tests due to the non-normal distribution of the data. Differences *p* < 0.05 between the group of untreated cells (negative control) and the group of cells treated with the compound were considered statistically significant according to the following criteria: * *p* < 0.05, ** *p* < 0.01, and *** *p* < 0.001. Statistical analysis of the data was performed using GraphPad Prism, version 5.00 (GraphPad Software, San Diego, CA, USA).

## Figures and Tables

**Table 1:** Cytotoxicity of unsymmetrical bisacridine derivatives (C-2028, C-2045, and C-2053) and positive control (gemcitabine—GEM or irinotecan—IR) against Panc-1, MIA PaCa-2, and AsPC-1 cells after 72 h of treatment. Data are expressed as IC_50_ and IC_80_ values, based on previously conducted studies on pancreatic cancer cells cultured in 2D [13].

Compound	Compound Dose [µM]	Cell Line		
		Panc-1	MIA PaCa-2	AsPC-1
C-2028	IC_80_	0.043 ± 0.002	0.042 ± 0.002	0.102 ± 0.007
C-2045	IC_80_	0.323 ± 0.043	0.086 ± 0.001	0.463 ± 0.011
C-2053	IC_80_	0.080 ± 0.012	0.072 ± 0.004	0.486 ± 0.005
GEM	IC_50_	not applicable	0.062 ± 0.010	0.026 ± 0.003
IR	IC_50_	8.5 ± 0.13	not applicable	not applicable

## Data Availability

Data obtained during this study are included in this article and the Supplementary Materials file. Detailed data are available from the corresponding author upon reasonable request.

## Supplementary Materials

The following supporting information can be downloaded at https://www.mdpi.com/article/10.3390/ijms26157557/s1.

## Abbreviations

The following abbreviations are used in this manuscript:

2D	Two-dimensional
3D	Three-dimensional
7-AAD	7-aminoactionomycin D
ABC	ATP-binding cassette
DMEM HG	High-glucose Dulbecco’s modified Eagle’s medium
DMSO	Dimethylsulfoxide
FACS	Fluorescence-Activated Cell Sorting
FOLFIRINOX	Multidrug: 5-fluorouracil, leucovorin, irinotecan and oxaliplatin
GEM	Gemcitabine
IR	Irinotecan
*KRAS*	Kirsten rat sarcoma virus gene
MCTS	Multicellular tumor spheroids
MDR	Multidrug resistance
NALIROX	Multidrug: 5-fluorouracil, leucovorin, liposomal irinotecan, and oxaliplatin
PBS	Phosphate-buffer saline
PC	Pancreatic cancer
PI	Propidium iodide
RPMI	Roswell Park Memorial Institute
RT	Room temperature
SMAD4	Mothers against decapentaplegic homolog 4
UAs	Unsymmetrical bisacridines
ULA	Ultra-low attachment

## References

[1] Quiñonero F., Mesas C., Doello K., Cabeza L., Perazzoli G., Jimenez-Luna C., Rosa Rama A., Melguizo C., Prados J. (2019). The Challenge of Drug Resistance in Pancreatic Ductal Adenocarcinoma: A Current Overview. Cancer Biol. Med..

[2] Jain A., Bhardwaj V. (2021). Therapeutic Resistance in Pancreatic Ductal Adenocarcinoma: Current Challenges and Future Opportunities. World J. Gastroenterol..

[3] National Cancer Institute Cancer Stat Facts: Pancreatic Cancer. https://seer.cancer.gov/statfacts/html/pancreas.html.

[4] Conroy T., Desseigne F., Ychou M., Bouché O., Guimbaud R., Bécouarn Y., Adenis A., Raoul J.-L., Gourgou-Bourgade S., de la Fouchardière C. (2011). FOLFIRINOX versus Gemcitabine for Metastatic Pancreatic Cancer. N. Engl. J. Med..

[5] Nichetti F., Rota S., Ambrosini P., Pircher C., Gusmaroli E., Droz Dit Busset M., Pusceddu S., Sposito C., Coppa J., Morano F. (2024). NALIRIFOX, FOLFIRINOX, and Gemcitabine With Nab-Paclitaxel as First-Line Chemotherapy for Metastatic Pancreatic Cancer. JAMA Netw. Open.

[6] Wang S., Li Y., Xing C., Ding C., Zhang H., Chen L., You L., Dai M., Zhao Y. (2020). Tumor Microenvironment in Chemoresistance, Metastasis and Immunotherapy of Pancreatic Cancer. Am. J. Cancer Res..

[7] Marin J., Monte M., Macias R., Romero M., Herraez E., Asensio M., Ortiz-Rivero S., Cives-Losada C., Di Giacomo S., Gonzalez-Gallego J. (2022). Expression of Chemoresistance-Associated ABC Proteins in Hepatobiliary, Pancreatic and Gastrointestinal Cancers. Cancers.

[8] Rajabpour A., Rajaei F., Teimoori-Toolabi L. (2017). Molecular Alterations Contributing to Pancreatic Cancer Chemoresistance. Pancreatology.

[9] Paluszkiewicz E., Horowska B., Borowa-Mazgaj B., Peszyńska-Sularz G., Paradziej-Łukowicz J., Augustin E., Konopa J., Mazerska Z. (2020). Design, Synthesis and High Antitumor Potential of New Unsymmetrical Bisacridine Derivatives towards Human Solid Tumors, Specifically Pancreatic Cancers and Their Unique Ability to Stabilize DNA G-Quadruplexes. Eur. J. Med. Chem..

[10] Laskowski T., Kosno M., Andrałojć W., Pakuła J., Stojałowski R., Borzyszkowska-Bukowska J., Paluszkiewicz E., Mazerska Z. (2025). The Interactions of Pu22 G-Quadruplex, Derived from c-MYC Promoter Sequence, with Antitumor Acridine Derivatives—An NMR/MD Combined Study. Mol. Ther. Nucleic Acids.

[11] Laskowski T., Kosno M., Andrałojć W., Frackowiak J.E., Borzyszkowska-Bukowska J., Szczeblewski P., Radoń N., Świerżewska M., Woźny A., Paluszkiewicz E. (2023). The Interactions of Monomeric Acridines and Unsymmetrical Bisacridines (UAs) with DNA Duplexes: An Insight Provided by NMR and MD Studies. Sci. Rep..

[12] Pawłowska M., Kulesza J., Augustin E. (2022). C-Myc Protein Level Affected by Unsymmetrical Bisacridines Influences Apoptosis and Senescence Induced in HCT116 Colorectal and H460 Lung Cancer Cells. Int. J. Mol. Sci..

[13] Kurdyn A., Pawłowska M., Paluszkiewicz E., Cichorek M., Augustin E. (2025). C-Myc Inhibition and P21 Modulation Contribute to Unsymmetrical Bisacridines-Induced Apoptosis and Senescence in Pancreatic Cancer Cells. Pharmacol. Rep..

[14] Kulesza J., Paluszkiewicz E., Augustin E. (2023). Cellular Effects of Selected Unsymmetrical Bisacridines on the Multicellular Tumor Spheroids of HCT116 Colon and A549 Lung Cancer Cells in Comparison to Monolayer Cultures. Int. J. Mol. Sci..

[15] Delle Cave D., Rizzo R., Sainz B., Gigli G., del Mercato L.L., Lonardo E. (2021). The Revolutionary Roads to Study Cell–Cell Interactions in 3D In Vitro Pancreatic Cancer Models. Cancers.

[16] Han S.J., Kwon S., Kim K.S. (2021). Challenges of Applying Multicellular Tumor Spheroids in Preclinical Phase. Cancer Cell Int..

[17] Mitrakas A.G., Tsolou A., Didaskalou S., Karkaletsou L., Efstathiou C., Eftalitsidis E., Marmanis K., Koffa M. (2023). Applications and Advances of Multicellular Tumor Spheroids: Challenges in Their Development and Analysis. Int. J. Mol. Sci..

[18] Minami F., Sasaki N., Shichi Y., Gomi F., Michishita M., Ohkusu-Tsukada K., Toyoda M., Takahashi K., Ishiwata T. (2021). Morphofunctional Analysis of Human Pancreatic Cancer Cell Lines in 2- and 3-Dimensional Cultures. Sci. Rep..

[19] Truong L.-H., Pauklin S. (2021). Pancreatic Cancer Microenvironment and Cellular Composition: Current Understandings and Therapeutic Approaches. Cancers.

[20] Deer E.L., González-Hernández J., Coursen J.D., Shea J.E., Ngatia J., Scaife C.L., Firpo M.A., Mulvihill S.J. (2010). Phenotype and Genotype of Pancreatic Cancer Cell Lines. Pancreas.

[21] Svirshchevskaya E., Doronina E., Grechikhina M., Matushevskaya E., Kotsareva O., Fattakhova G., Sapozhnikov A., Felix K. (2019). Characteristics of Multicellular Tumor Spheroids Formed by Pancreatic Cells Expressing Different Adhesion Molecules. Life Sci..

[22] Shichi Y., Gomi F., Sasaki N., Nonaka K., Arai T., Ishiwata T. (2022). Epithelial and Mesenchymal Features of Pancreatic Ductal Adenocarcinoma Cell Lines in Two- and Three-Dimensional Cultures. J. Pers. Med..

[23] Kopantzev E.P., Kopantseva M.R., Grankina E.V., Mikaelyan A., Egorov V.I., Sverdlov E.D. (2019). Activation of IGF/IGF-IR Signaling Pathway Fails to Induce Epithelial-Mesenchymal Transition in Pancreatic Cancer Cells. Pancreatology.

[24] Ungefroren H., Thürling I., Färber B., Kowalke T., Fischer T., De Assis L.V.M., Braun R., Castven D., Oster H., Konukiewitz B. (2022). The Quasimesenchymal Pancreatic Ductal Epithelial Cell Line PANC-1—A Useful Model to Study Clonal Heterogeneity and EMT Subtype Shifting. Cancers.

[25] Rice A.J., Cortes E., Lachowski D., Cheung B.C.H., Karim S.A., Morton J.P., del Río Hernández A. (2017). Matrix Stiffness Induces Epithelial–Mesenchymal Transition and Promotes Chemoresistance in Pancreatic Cancer Cells. Oncogenesis.

[26] Liao Q., Hu Y., Zhao Y.-P., Zhou T., Zhang Q. (2010). Assessment of Pancreatic Carcinoma Cell Chemosensitivity Using a Three-Dimensional Culture System. Chin. Med. J. (Engl.).

[27] Wen Z., Liao Q., Hu Y., You L., Zhou L., Zhao Y. (2013). A Spheroid-Based 3-D Culture Model for Pancreatic Cancer Drug Testing, Using the Acid Phosphatase Assay. Braz. J. Med. Biol. Res..

[28] Singh S.K., Abbas S., Saxena A.K., Tiwari S., Sharma L.K., Tiwari M. (2020). Critical Role of Three-Dimensional Tumorsphere Size on Experimental Outcome. Biotechniques.

[29] Alwahsh M., Al-Doridee A., Jasim S., Awwad O., Hergenröder R., Hamadneh L. (2024). Cytotoxic and Molecular Differences of Anticancer Agents on 2D and 3D Cell Culture. Mol. Biol. Rep..

[30] Paškevičiūtė M., Petrikaitė V. (2017). Differences of Statin Activity in 2D and 3D Pancreatic Cancer Cell Cultures. Drug Des. Devel. Ther..

[31] Tidwell T.R., Røsland G., Tronstad K.J., Søreide K., Hagland H.R. (2024). Comparing in Vitro Cytotoxic Drug Sensitivity in Colon Andpancreatic Cancer Using 2D and 3D Cell Models: Contrastingviability and Growth Inhibition in Clinically Relevant Doseand Repeated Drug Cycles. Cancer Med..

[32] Breslin S., O’Driscoll L. (2016). The Relevance of Using 3D Cell Cultures, in Addition to 2D Monolayer Cultures, When Evaluating Breast Cancer Drug Sensitivity and Resistance. Oncotarget.

[33] Ascione F., Ferraro R., Dogra P., Cristini V., Guido S., Caserta S. (2024). Gradient-Induced Instability in Tumour Spheroids Unveils the Impact of Microenvironmental Nutrient Changes. Sci. Rep..

[34] Lee J.-H., Kim S.-K., Khawar I.A., Jeong S.-Y., Chung S., Kuh H.-J. (2018). Microfluidic Co-Culture of Pancreatic Tumor Spheroids with Stellate Cells as a Novel 3D Model for Investigation of Stroma-Mediated Cell Motility and Drug Resistance. J. Exp. Clin. Cancer Res..

[35] Jaros S.W., Komarnicka U.K., Kyzioł A., Pucelik B., Nesterov D.S., Kirillov A.M., Smoleński P. (2022). Therapeutic Potential of a Water-Soluble Silver-Diclofenac Coordination Polymer on 3D Pancreatic Cancer Spheroids. J. Med. Chem..

[36] Achilli T.-M., McCalla S., Meyer J., Tripathi A., Morgan J.R. (2014). Multilayer Spheroids To Quantify Drug Uptake and Diffusion in 3D. Mol. Pharm..

[37] Hagmann W., Faissner R., Schnölzer M., Löhr M., Jesnowski R. (2010). Membrane Drug Transporters and Chemoresistance in Human Pancreatic Carcinoma. Cancers.

[38] Harpstrite S.E., Gu H., Natarajan R., Sharma V. (2014). Interrogation of Multidrug Resistance (MDR1) P-Glycoprotein (ABCB1) Expression in Human Pancreatic Carcinoma Cells. Nucl. Med. Commun..

[39] Pawłowska M., Kulesza J., Paluszkiewicz E., Augustin E., Mazerska Z. (2024). Unsymmetrical Bisacridines’ Interactions with ABC Transporters and Their Cellular Impact on Colon LS 174T and Prostate DU 145 Cancer Cells. Molecules.

[40] Hassan S., Peluso J., Chalhoub S., Idoux Gillet Y., Benkirane-Jessel N., Rochel N., Fuhrmann G., Ubeaud-Sequier G. (2020). Quercetin Potentializes the Respective Cytotoxic Activity of Gemcitabine or Doxorubicin on 3D Culture of AsPC-1 or HepG2 cells, through the inhibition of HIF-1α and MDR1. PLoS ONE.

[41] Tidwell T.R., Røsland G.V., Tronstad K.J., Søreide K., Hagland H.R. (2022). Metabolic Flux Analysis of 3D Spheroids Reveals Significant Differences in Glucose Metabolism from Matched 2D Cultures of Colorectal Cancer and Pancreatic Ductal Adenocarcinoma Cell Lines. Cancer Metab..

[42] Pilch J., Potęga A., Kowalczyk A., Kasprzak A., Kowalik P., Bujak P., Paluszkiewicz E., Augustin E., Nowicka A.M. (2023). PH-Responsive Drug Delivery Nanoplatforms as Smart Carriers of Unsymmetrical Bisacridines for Targeted Cancer Therapy. Pharmaceutics.

[43] Patki M., Saraswat A., Bhutkar S., Dukhande V., Patel K. (2021). In Vitro Assessment of a Synergistic Combination of Gemcitabine and Zebularine in Pancreatic Cancer Cells. Exp. Cell Res..

[44] Samanta K., Setua S., Kumari S., Jaggi M., Yallapu M.M., Chauhan S.C. (2019). Gemcitabine Combination Nano Therapies for Pancreatic Cancer. Pharmaceutics.

