# Supplementary material for: Pro-Apoptotic Effects of Unsymmetrical Bisacridines in 3D Pancreatic Multicellular Tumor Spheroids
Source: Int J Mol Sci. 2025 Aug 5;26(15):7557. doi: 10.3390/ijms26157557 (PMC12347750; doi:10.3390/ijms26157557)
Supplement: Supplementary file 1 [file ijms-26-07557-s001.zip › ijms-3787417-supplementary.pdf]

International Journal of Molecular Sciences

MDPI

Supplementary Materials

Pro-Apoptotic Effects of Unsymmetrical Bisacridines in 3D Pancreatic Multicellular Tumor Spheroids

Agnieszka Kurdyn, Ewa Paluszkiewicz and Ewa Augustin *

Department of Pharmaceutical Technology and Biochemistry, Faculty of Chemistry, Gdańsk University of Technology, Gabriela Narutowicza Str. 11/12, 80-233 Gdańsk, Poland
* Correspondence: ewa.augustin@pg.edu.pl; Tel.: +48-58-347-14-68

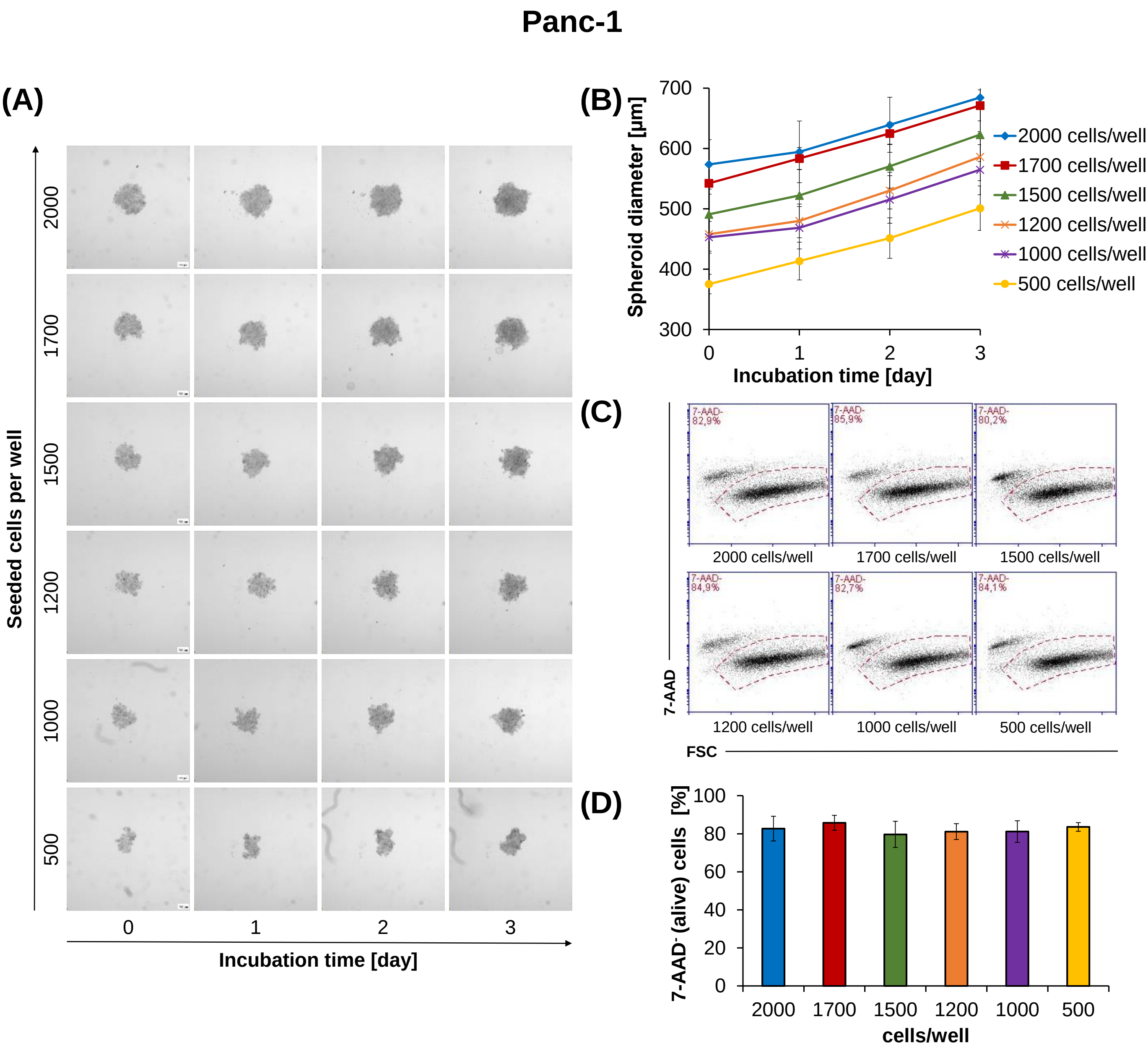

Figure S1 Establishment of seeding conditions for Panc-1 spheroids. (A) Representative images of spheroids generated from different cell numbers. (B) Growth curves of the spheroids. (C) Representative cytograms of cell viability analysis and (d) the bar graph with the mean percentage of alive cells at day 3. Scale bar 200 µm. (n≥3)

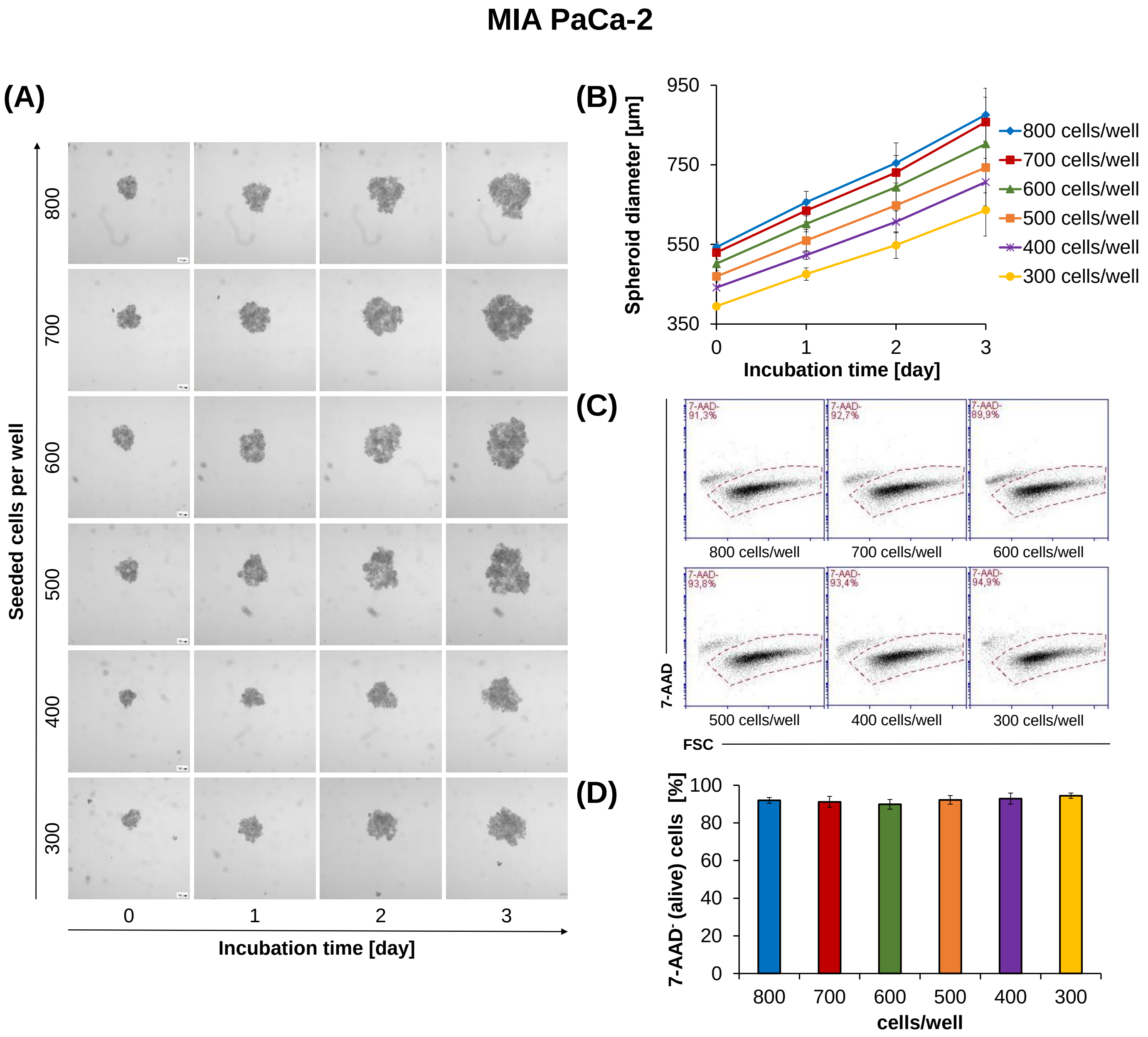

Figure S2 Establishment of seeding conditions for MIA PaCa-2 spheroids. (A) Representative images of spheroids generated from different cell numbers. (B) Growth curves of the spheroids. (C) Representative cytograms of cell viability analysis and (d) the bar graph with the mean percentage of alive cells at day 3. Scale bar 200 µm. (n≥3)

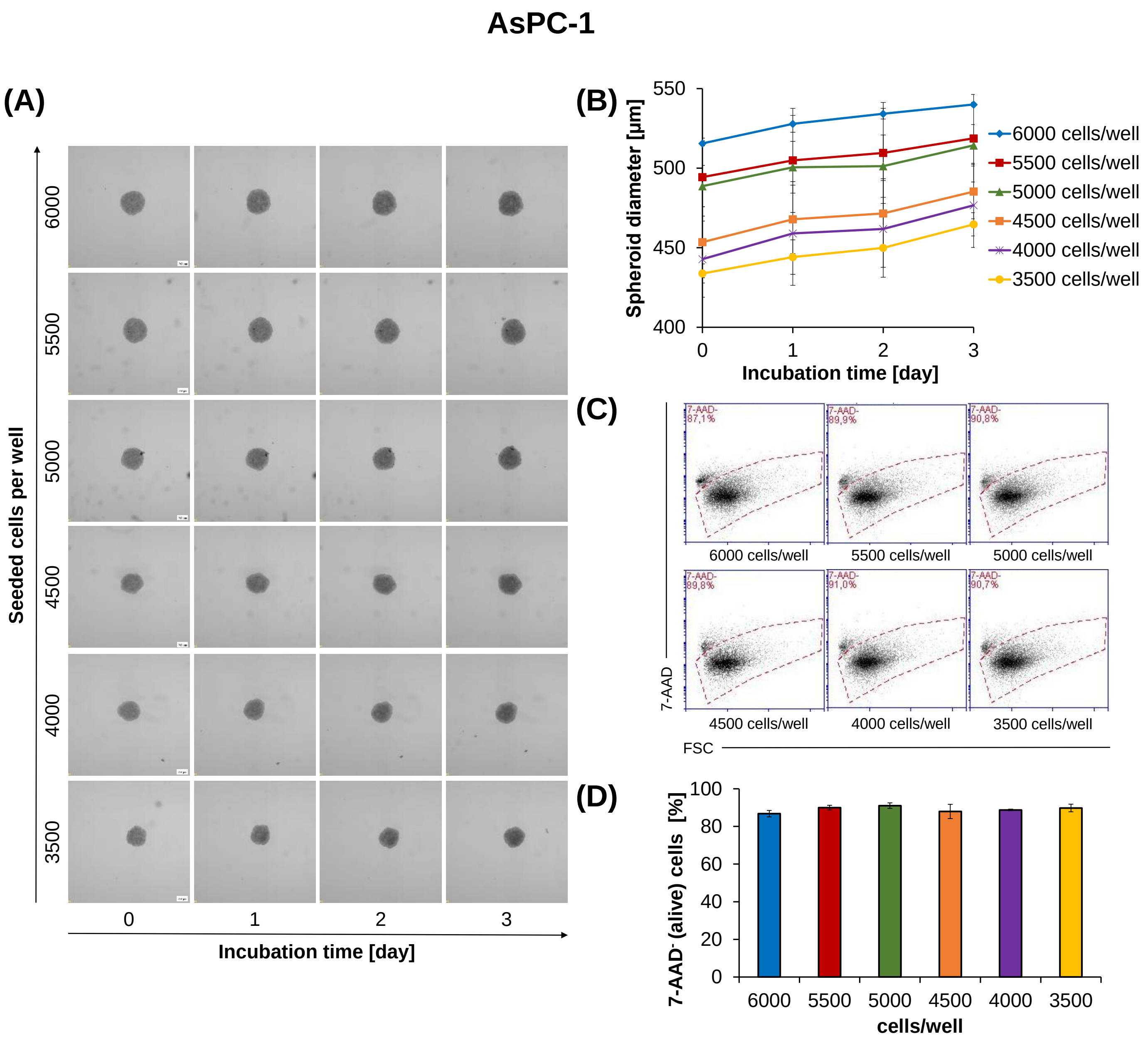

Figure S3 Establishment of seeding conditions for AsPC-1 spheroids. (A) Representative images of spheroids generated from different cell numbers. (B) Growth curves of the spheroids. (C) Representative cytograms of cell viability analysis and (d) the bar graph with the mean percentage of alive cells at day 3. Scale bar 200 µm. (n≥3)

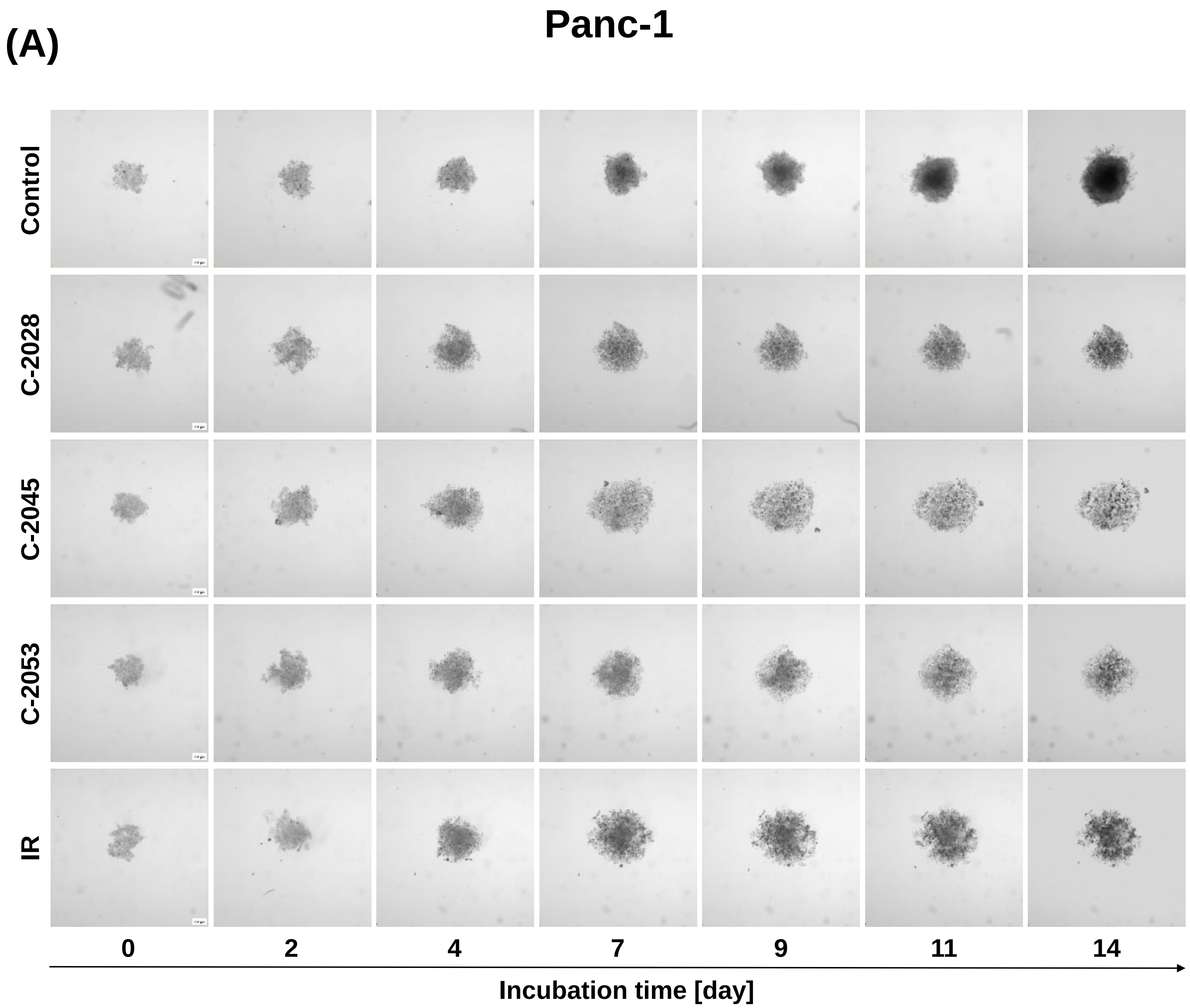

(B)

Panc-1									
Spheroid growth [%]			Incubation time [day]						
			**0**	**2**	**4**	**7**	**9**	**11**	**14**
	**Control**	**mean**	**0**	**16**	**31**	**50**	**62**	**74**	**90**
		SD	0	2	2	3	5	5	7
	**C-2028**	**mean**	**0**	**17**	**27**	**35**	**29**	**25**	**17**
		SD	0	4	3	4	7	2	5
	**C-2045**	**mean**	**0**	**23**	**32**	**57**	**58**	**48**	**35**
		SD	0	2	5	5	2	7	4
	**C-2053**	**mean**	**0**	**20**	**32**	**34**	**33**	**45**	**29**
		SD	0	5	4	7	4	6	5
	**IR**	**mean**	**0**	**9**	**20**	**44**	**51**	**52**	**47**
		SD	0	4	4	4	5	6	7

Figure S4 Panc-1 spheroids (A) morphology and (B) growth kinetics after treatment with UAs at IC_{80} and irinotecan (IR) at IC_{50}. Scale bar 200 µm. (n=4)

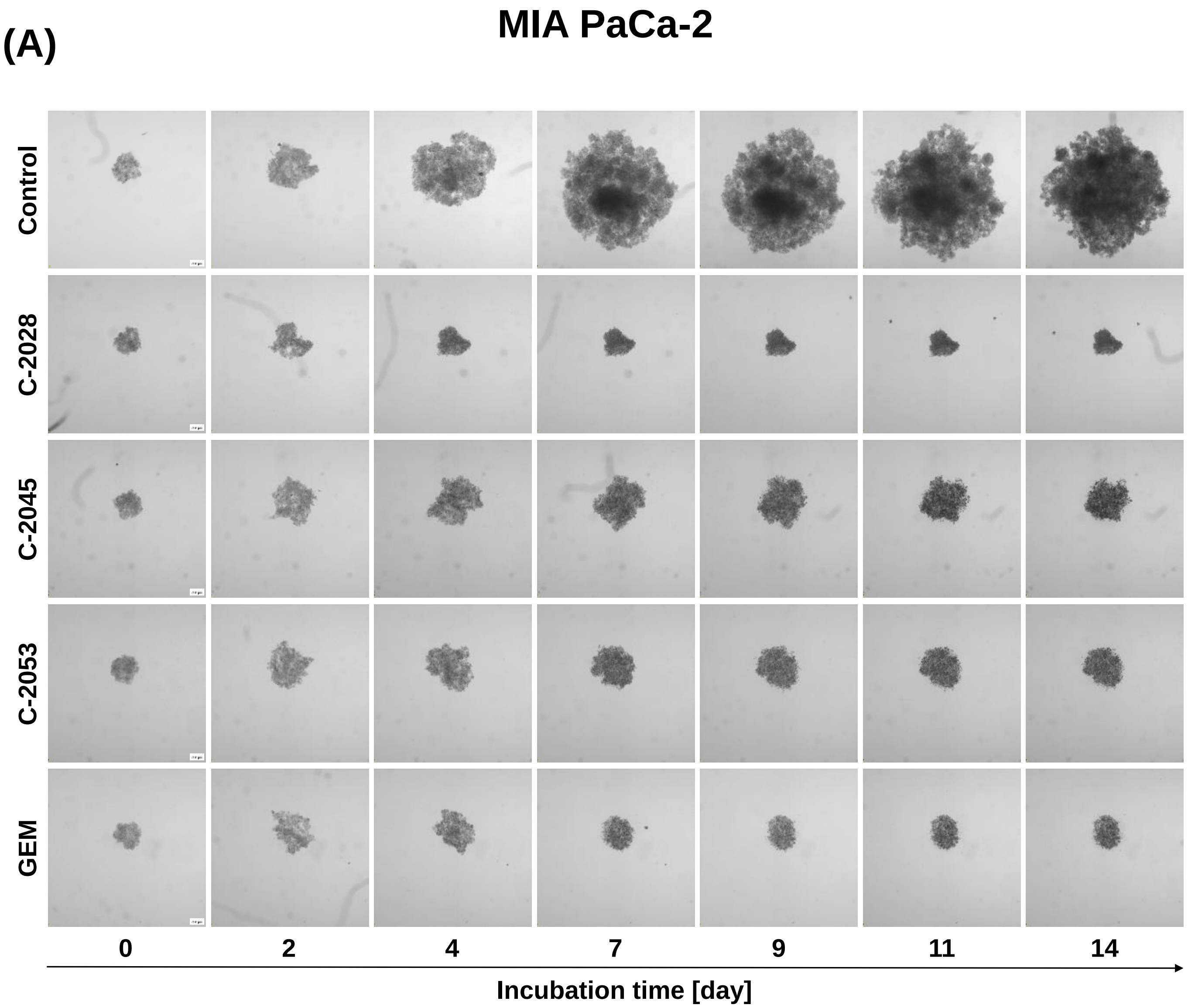

(B)

MIA PaCa-2									
Spheroid growth [%]			Incubation time [day]						
			0	2	4	7	9	11	14
	Control	mean	**0**	**47**	**128**	**248**	**313**	**326**	**338**
		SD	0	3	11	22	6	19	7
	C-2028	mean	**0**	**33**	**21**	**15**	**11**	**8**	**5**
		SD	0	3	4	2	2	2	3
	C-2045	mean	**0**	**45**	**71**	**86**	**86**	**87**	**63**
		SD	0	7	4	4	3	3	3
	C-2053	mean	**0**	**41**	**56**	**51**	**46**	**41**	**35**
		SD	0	7	6	7	7	5	5
	GEM	mean	**0**	**30**	**36**	**23**	**19**	**19**	**12**
		SD	0	6	2	4	3	5	4

Figure S5 MIA PaCa-2 spheroids (A) morphology and (B) growth kinetics after treatment with UAs at IC_{80} and gemcitabine (GEM) at IC_{50}. Scale bar 200 µm. (n=4)

(A) **AsPC-1**

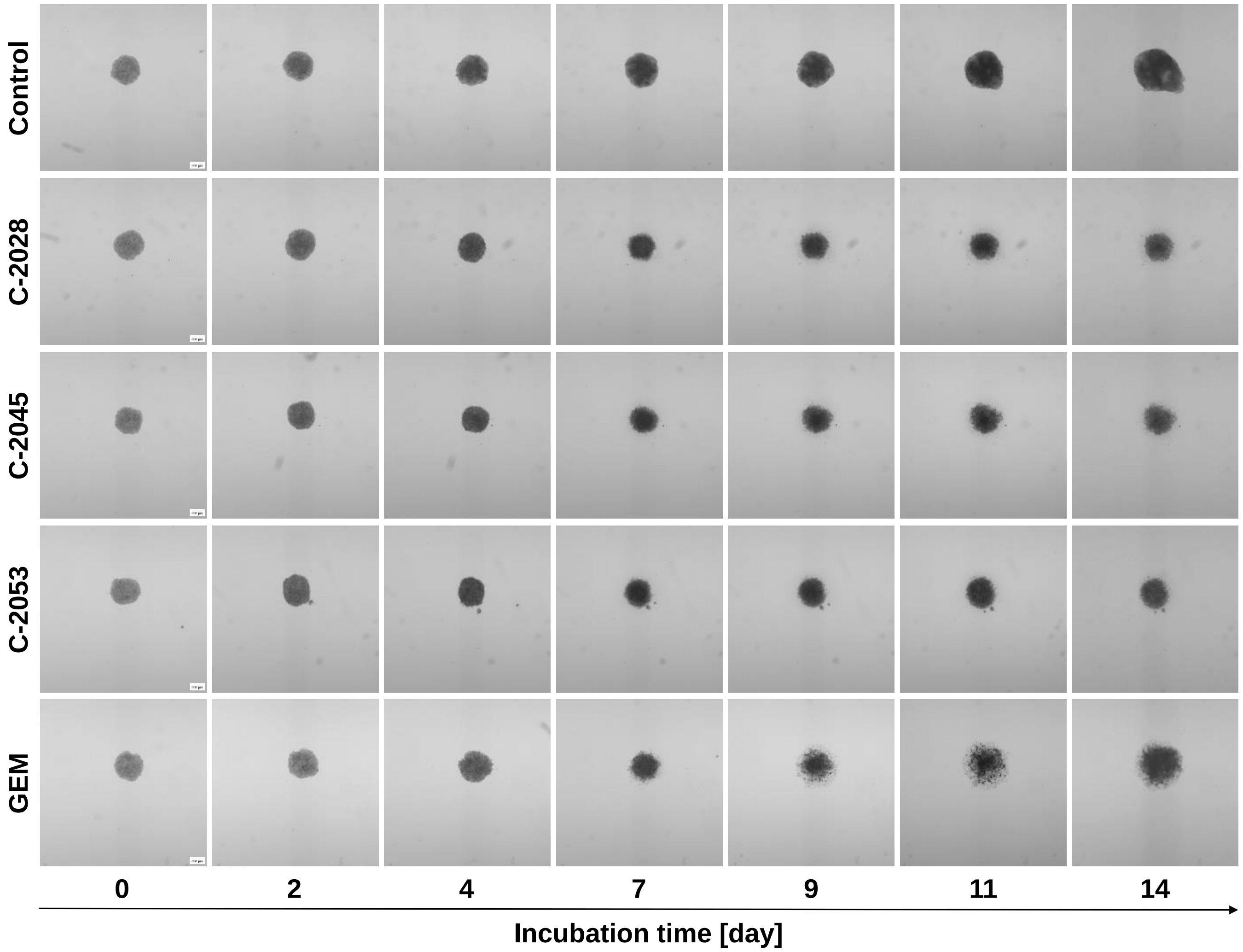

(B)

AsPC-1									
			Incubation time [day]						
			0	2	4	7	9	11	14
Spheroid growth [%]	Control	mean	**0**	**3**	**10**	**25**	**35**	**51**	**73**
		SD	0	1	2	1	2	3	3
	C-2028	mean	**0**	**0**	**0**	**-2**	**-2**	**3**	**11**
		SD	0	4	3	1	1	3	6
	C-2045	mean	**0**	**2**	**2**	**1**	**-1**	**5**	**18**
		SD	0	2	2	2	4	2	4
	C-2053	mean	**0**	**-1**	**-1**	**-1**	**0**	**4**	**7**
		SD	0	4	1	4	3	1	2
	GEM	mean	**0**	**2**	**3**	**-4**	**6**	**20**	**30**
		SD	0	2	3	4	3	1	2

Figure S6 AsPC-1 spheroids (A) morphology and (B) growth kinetics after treatment with UAs at IC_{80} and gemcitabine (GEM) at IC_{50}. Scale bar 200 μm. (n=4)

Table S1 Cell viability of Panc-1, Mia PaCa-2, and AsPC-1 cells after 72h of treatment with UAs at IC_{80} and reference compounds (IR – irinotecan, GEM – gemcitabine) at IC_{50} cultured in 2D conditions. (n=4)

2D cell culture							
		Panc-1		MIA PaCa-2		AsPC-1	
		mean	±	mean	±	mean	±
Control	live (7-AAD$^-$)	**92,3**	1,1	**93,6**	1,9	**95,1**	0,4
	dead (7-AAD$^+$)	**7,8**	1,1	**6,4**	1,9	**4,9**	0,4
C-2028	live (7-AAD$^-$)	**45,6**	6,9	**38,2**	7,1	**62,3**	1,5
	dead (7-AAD$^+$)	**54,5**	6,9	**61,8**	7,1	**37,7**	1,5
C-2045	live (7-AAD$^-$)	**39,6**	3,3	**61,2**	3,5	**59,2**	5,6
	dead (7-AAD$^+$)	**60,4**	3,3	**38,8**	3,5	**40,9**	5,6
C-2053	live (7-AAD$^-$)	**51,3**	4,2	**64,4**	1,8	**53,9**	3,8
	dead (7-AAD$^+$)	**48,7**	4,2	**35,6**	1,8	**46,1**	3,8
IR	live (7-AAD$^-$)	**51,4**	6,8	-	-	-	-
	dead (7-AAD$^+$)	**48,6**	6,8	-	-	-	-
GEM	live (7-AAD$^-$)	-	-	**60,2**	6,4	**59,4**	5,2
	dead (7-AAD$^+$)	-	-	**39,8**	6,4	**40,6**	5,2

Table S2 Cell viability of Panc-1, Mia PaCa-2, and AsPC-1 cells after 72h of treatment with UAs at IC_{80} and reference compounds (IR – irinotecan, GEM – gemcitabine) at IC_{50} cultured in 3D conditions. (n=4)

3D cell culture							
		**Panc-1**		**MIA PaCa-2**		**AsPC-1**	
		**mean**	**±**	**mean**	**±**	**mean**	**±**
**Control**	live (7-AAD$^-$)	**85,3**	3,1	**90,4**	2,4	**90,3**	1,7
	dead (7-AAD$^+$)	**14,7**	3,1	**8,7**	2,4	**9,7**	1,7
**C-2028**	live (7-AAD$^-$)	**56,2**	4,3	**60,6**	7,1	**78,3**	6,1
	dead (7-AAD$^+$)	**43,8**	4,3	**39,4**	7,1	**21,7**	6,1
**C-2045**	live (7-AAD$^-$)	**54,0**	6,0	**81,3**	3,8	**77,5**	8,6
	dead (7-AAD$^+$)	**48,7**	6,0	**18,7**	3,8	**22,5**	8,6
**C-2053**	live (7-AAD$^-$)	**65,7**	5,3	**78,1**	6,4	**73,9**	8,0
	dead (7-AAD$^+$)	**34,3**	5,3	**21,9**	6,4	**26,1**	8,0
**IR**	live (7-AAD$^-$)	**66,3**	11,5	-	-	-	-
	dead (7-AAD$^+$)	**33,7**	11,5	-	-	-	-
**GEM**	live (7-AAD$^-$)	-	-	**74,3**	8,5	**78,6**	4,9
	dead (7-AAD$^+$)	-	-	**25,8**	8,5	**21,4**	4,9

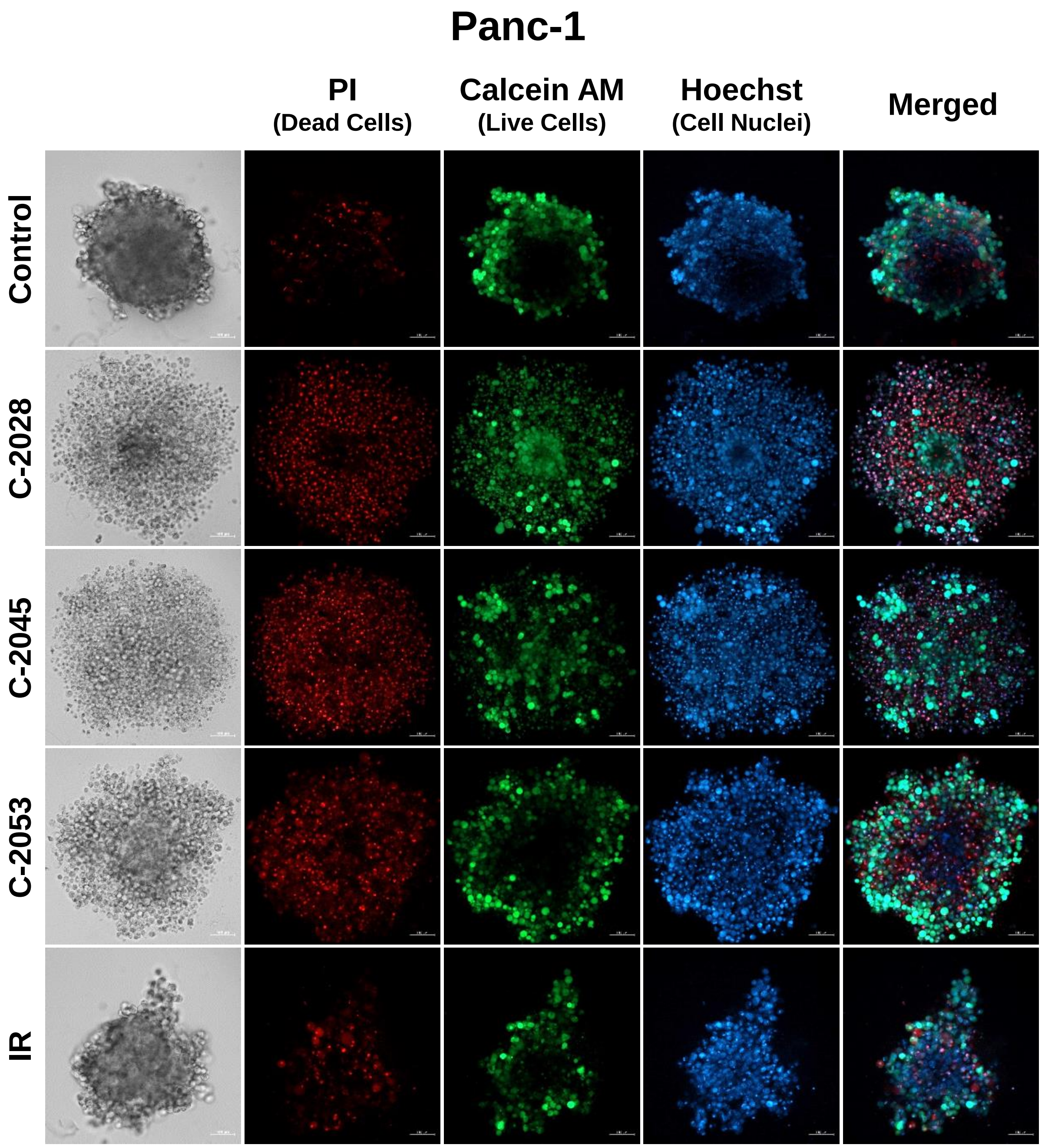

Figure S7 Representative images of Panc-1 spheroids after 72h of treatment with UAs at IC_{80} and irinotecan (IR) at IC_{50}. Spheroids were stained with PI, Calcein AM, and Hoechst 33342. Scale bar 100 µm. (n = 2)

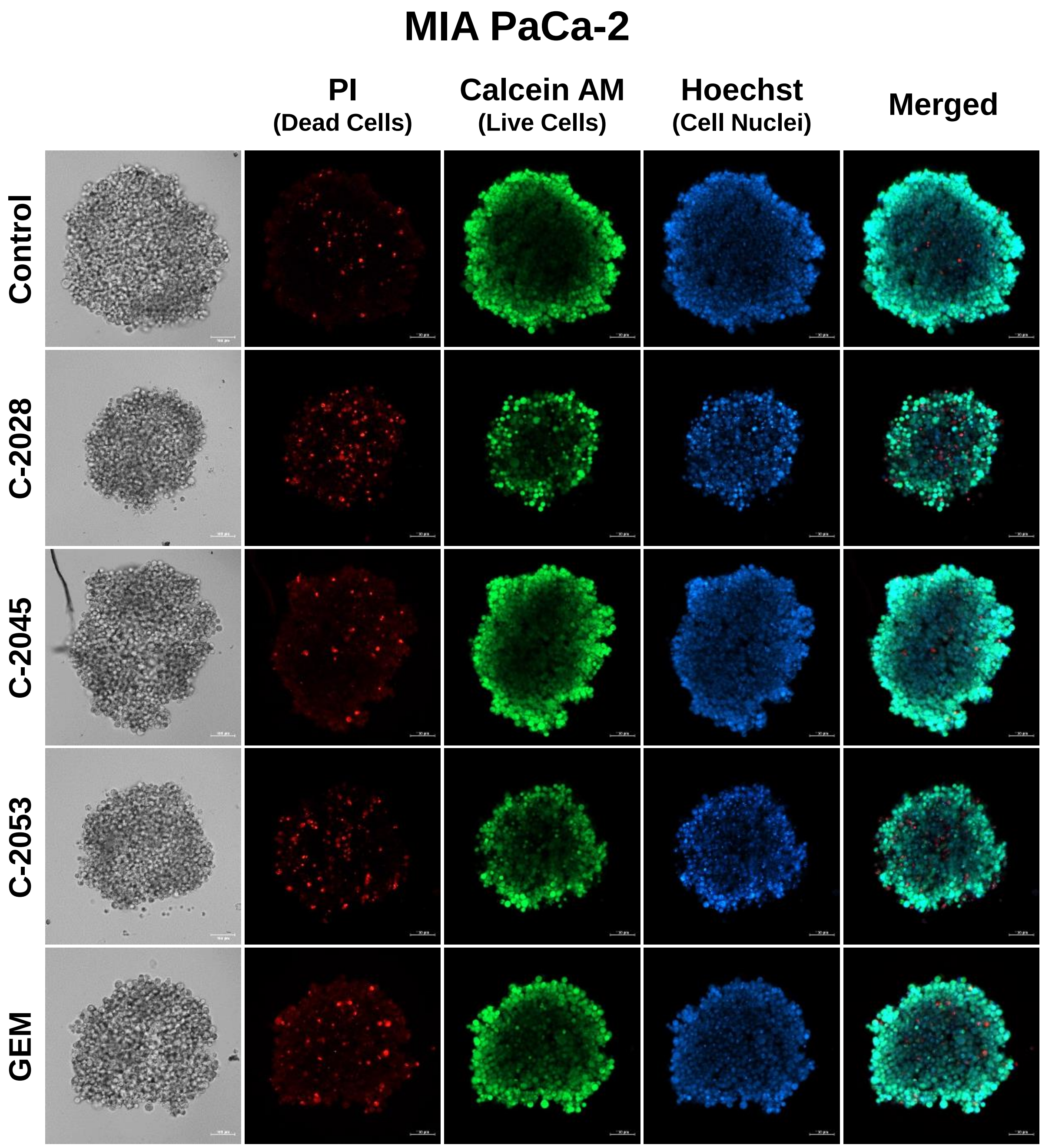

Figure S8 Representative images of MIA PaCa-2 spheroids after 72h of treatment with UAs at IC_{80} and gemcitabine (GEM) at IC_{50}. Spheroids were stained with PI, Calcein AM, and Hoechst 33342. Scale bar 100 μm. (n = 2)

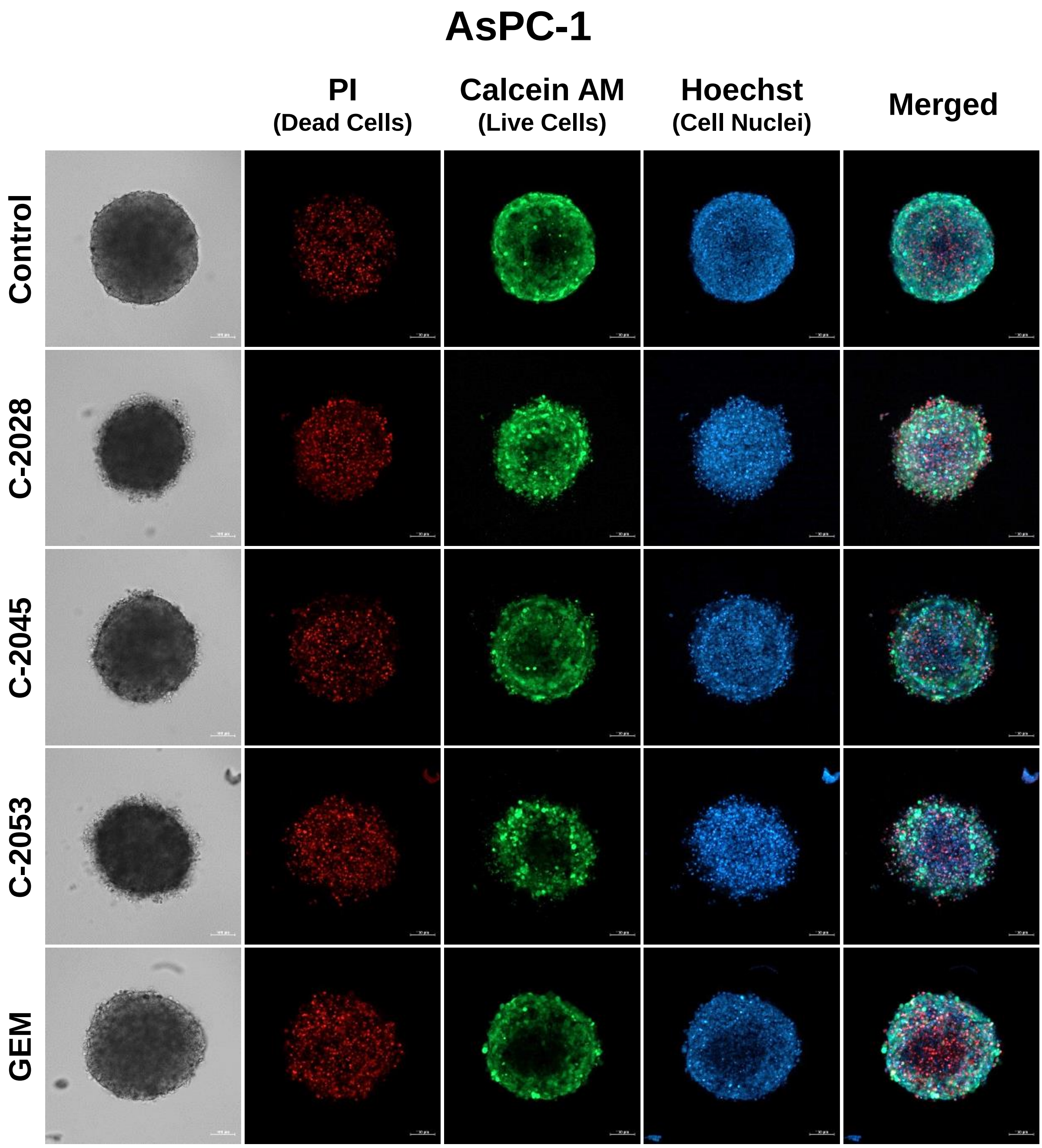

Figure S9 Representative images of AsPC-1 spheroids after 72h of treatment with UAs at IC_{80} and gemcitabine (GEM) at IC_{50}. Spheroids were stained with PI, Calcein AM, and Hoechst 33342. Scale bar 100 µm. (n = 2)

Table S3 Analysis of phosphatidylserine externalization and membrane disruption in Panc-1, MIA PaCa-2, and AsPC-1 cells after 72h of treatment with UAs at IC_{80} doses and and reference compounds (IR – irinotecan, GEM – gemcitabine) at IC_{50} cultured in 3D conditions. (n=4)

3D cell culture							
		Panc-1		MIA		AsPC-1	
		mean	SD	mean	SD	mean	SD
Control	A-/PI-	**79,1**	0,2	**86,9**	1,5	**84,6**	3,1
	A+/PI-	**11,3**	1,8	**6,3**	1,4	**7,8**	3,1
	A+/PI+	**6,8**	1,1	**5,8**	0,7	**6,0**	1,9
	A-/PI+	**2,6**	0,6	**1,0**	0,5	**2,0**	0,6
C-2028	A-/PI-	**44,0**	4,4	**55,3**	3,2	**73,5**	2,1
	A+/PI-	**29,2**	7,4	**28,0**	4,2	**11,7**	0,8
	A+/PI+	**24,0**	6,0	**13,0**	3,4	**9,8**	2,7
	A-/PI+	**2,8**	1,6	**3,8**	1,8	**5,1**	2,0
C-2045	A-/PI-	**45,1**	6,0	**67,6**	6,3	**71,4**	6,7
	A+/PI-	**34,3**	4,6	**14,0**	4,4	**10,8**	1,9
	A+/PI+	**20,1**	4,1	**12,7**	2,9	**12,4**	4,8
	A-/PI+	**4,3**	1,1	**5,8**	2,8	**5,5**	2,4
C-2053	A-/PI-	**60,9**	1,7	**61,1**	8,3	**76,5**	3,6
	A+/PI-	**18,5**	2,4	**19,2**	4,0	**10,8**	2,2
	A+/PI+	**13,5**	2,7	**15,0**	3,9	**9,6**	2,8
	A-/PI+	**7,2**	0,9	**4,7**	1,6	**3,2**	0,8
IR	A-/PI-	**61,2**	4,1	-	-	-	-
	A+/PI-	**16,2**	3,1	-	-	-	-
	A+/PI+	**13,2**	3,6	-	-	-	-
	A-/PI+	**9,5**	2,1	-	-	-	-
GEM	A-/PI-	-	-	**65,8**	4,3	**72,7**	2,4
	A+/PI-	-	-	**11,7**	1,4	**12,8**	1,7
	A+/PI+	-	-	**13,8**	2,5	**10,9**	2,6
	A-/PI+	-	-	**7,5**	4,4	**3,6**	0,8

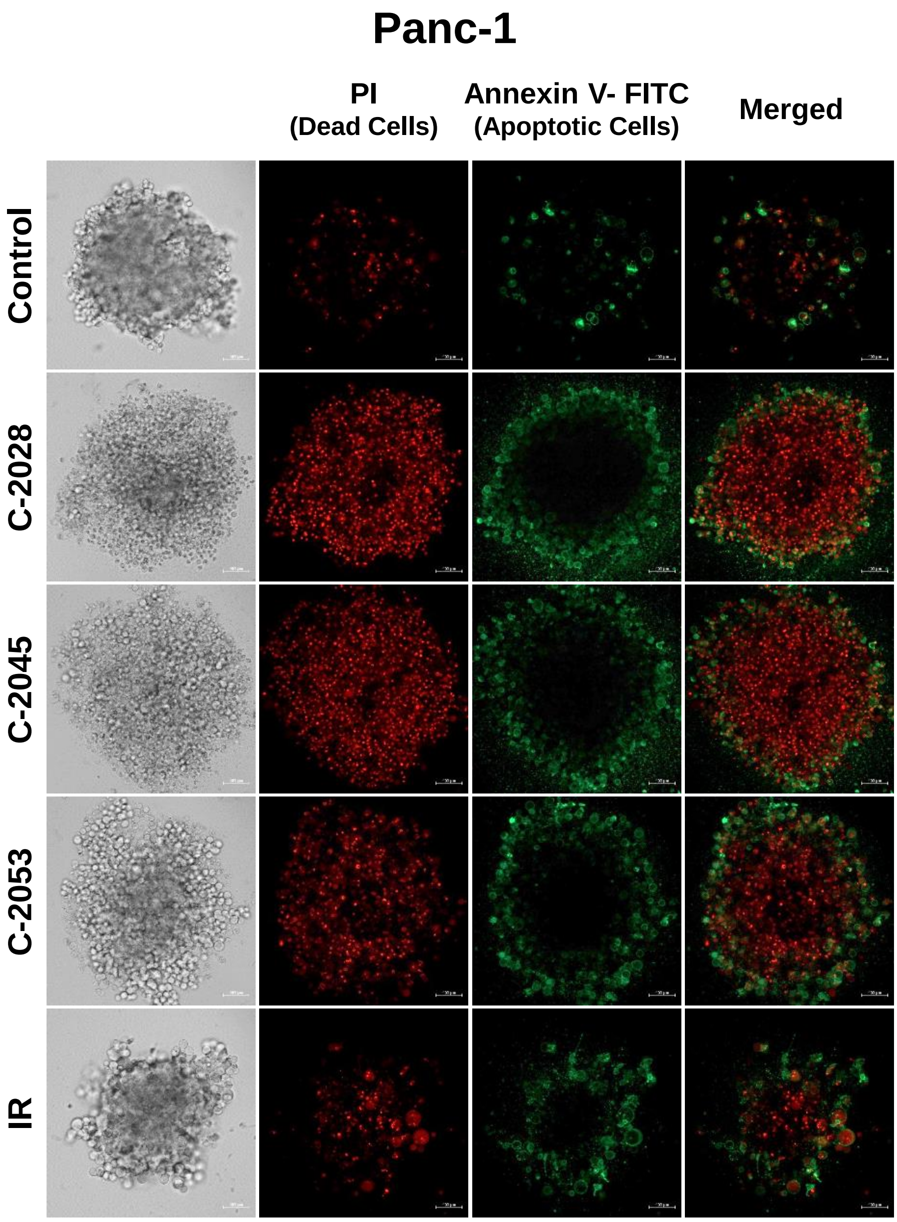

Figure S10 Representative images of Panc-1 spheroids after 72h of treatment with UAs at IC_{80} and irinotecan (IR) at IC_{50}. Spheroids were stained with PI and annexin V-FITC. Scale bar 100 µm. (n = 2)

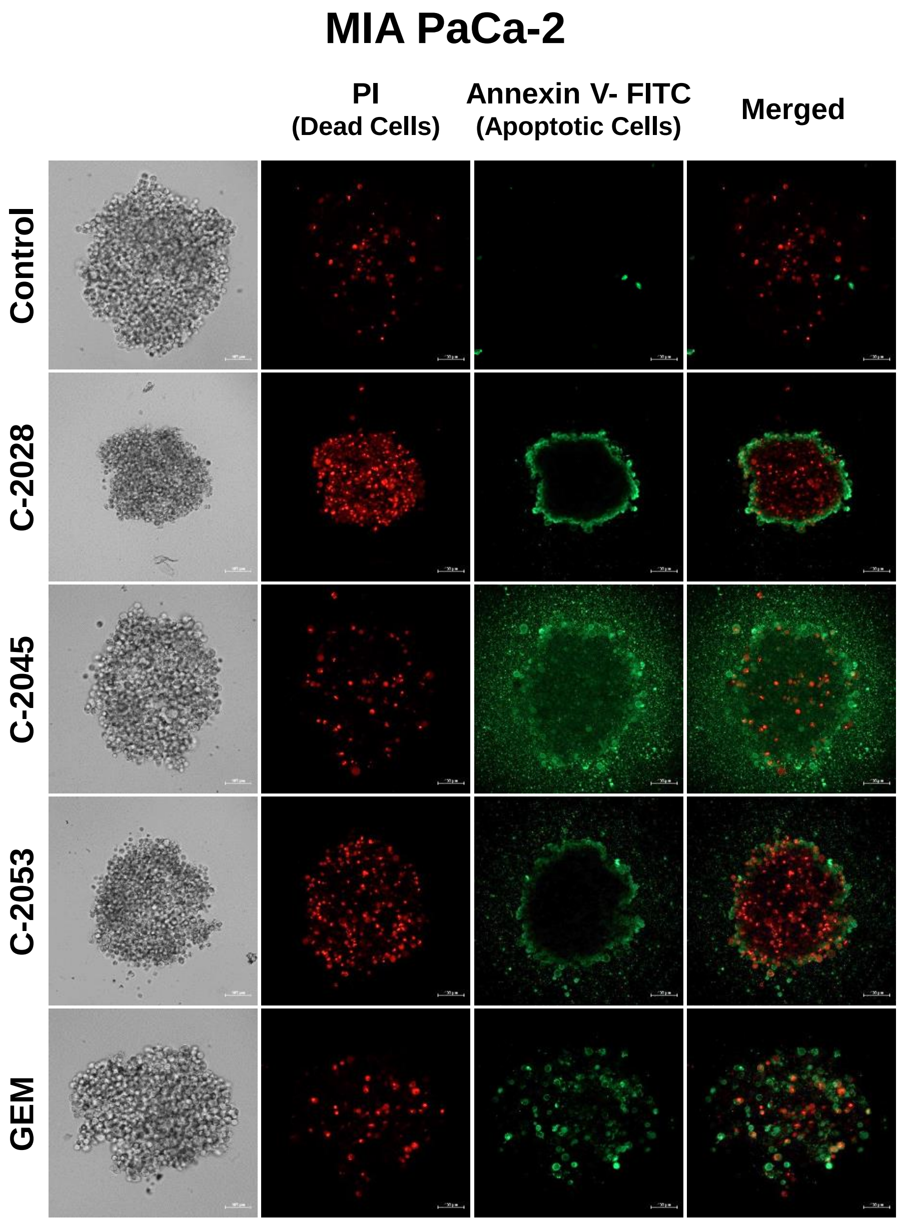

FigureS11 Representative images of MIA PaCa-2 spheroids after 72h of treatment with UAs at IC_{80} and gemcitabine (GEM) at IC_{50}. Spheroids were stained with PI and annexin V-FITC. Scale bar 100 µm. (n = 2)

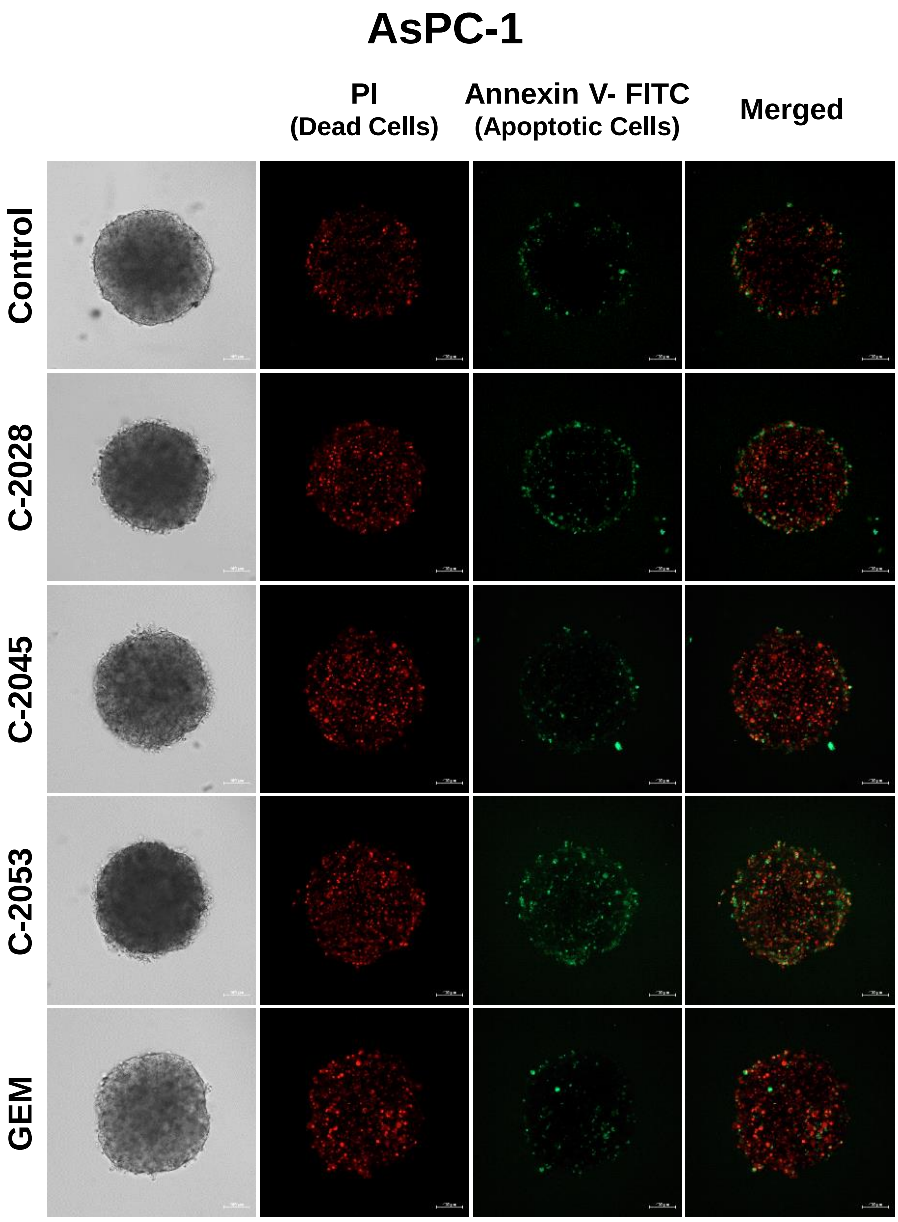

Figure S12 Representative images of AsPC-1 spheroids after 72h of treatment with UAs at IC_{80} and gemcitabine (GEM) at IC_{50}. Spheroids were stained with PI and annexin V-FITC. Scale bar 100 µm. (n = 2)
